# Diabetic Wound Repair: From Mechanism to Therapeutic Opportunities

**DOI:** 10.1002/mco2.70406

**Published:** 2025-09-29

**Authors:** Renyuan Wang, Song Gu, Young Hwa Kim, Aejin Lee, Haodong Lin, Dongsheng Jiang

**Affiliations:** ^1^ Precision Research Center For Refractory Diseases, Shanghai General Hospital Shanghai Jiao Tong University School of Medicine Shanghai China; ^2^ Trauma Center, Shanghai General Hospital Shanghai Jiao Tong University School of Medicine Shanghai China; ^3^ Inflamm‐Aging Translational Research Center Ajou University Medical Center Suwon Republic of Korea; ^4^ Food and Nutrition Major, Division of Integrative Biosciences Myongji University Yongin Republic of Korea; ^5^ Shanghai Jiao Tong University Pioneer Research Institute For Molecular and Cell Therapies, State Key Laboratory of Innovative Immunotherapy, School of Pharmacy, Shanghai Jiao Tong University Shanghai China

**Keywords:** animal models, cellular dysfunction, diabetic wounds, emerging therapies, metabolic microenvironment

## Abstract

Diabetic wound healing, characterized by persistent inflammation, impaired angiogenesis, and dysfunctional cellular responses, remains a major clinical challenge due to its complex pathophysiology. This challenge is most evident in diabetic foot ulcers (DFUs), which carry high risks of infection, recurrence, and amputation, contributing substantially to patient morbidity, mortality, and healthcare costs. Despite multidisciplinary care, debridement, and advanced dressings, healing outcomes are often suboptimal, highlighting an urgent need for deeper pathophysiological insights and more effective therapeutic strategies. This review synthesizes current understanding of DFU pathogenesis, emphasizing how sustained metabolic dysfunction disrupts fibroblast and immune cell function, thereby perpetuating chronic wounds. We also critically examine commonly used animal models and their limitations in replicating the complexity of human DFUs and discuss emerging therapeutic approaches with translational promise. Advancing our understanding of these mechanisms and validating innovative interventions may ultimately reduce DFU‐related amputations and mortality, improve healing outcomes, and enhance patient quality of life. This review aims to catalyze future research and therapeutic innovation in diabetic wound care.

## Graphical abstract

Diabetic wound healing is compromised by chronic metabolic dysfunction that disrupts essential cellular processes. This review examines key predisposing factors, including neuropathy and vascular disease, characterizes dysfunction in critical cell types such as macrophages and fibroblasts, evaluates limitations of current models, compares established and emerging therapies, and outlines future research and clinical strategies to advance management for diabetic foot ulcers.

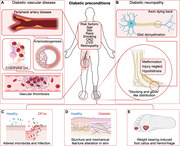

## 1 Introduction

The skin, the largest organ of the human body, performs vital roles in protection, sensation, thermoregulation, metabolism, excretion, and immune regulation [1]. It serves as the primary barrier against chemical, physical, microbial, and ultraviolet challenges [2]. Disruption of this barrier due to injuries from accidents or surgeries leads to skin wounds, necessitating timely and effective repair to restore integrity and function. Wound healing depends on a coordinated interplay of self‐renewal and homeostasis, influenced by factors such as nutrition, age, disease, and medications [3, 4, 5, 6]. Delayed wound healing results in chronic wounds, which increase the risk of infection, persistent pain, restricted mobility, and diminished quality of life, often leading to psychological distress [7]. Chronic wounds are generally defined as skin defects that fail to heal within 3 months, though their duration and severity vary among patients [8].

Diabetes mellitus is a leading cause of chronic wounds. This metabolic disorder, characterized by hyperglycemia [9], predisposes individuals to diabetic foot ulcers (DFUs), severe complications arising from minor skin injuries. Given their complexity, DFUs are defined by the International Working Group on the Diabetic Foot in the 2023 update as “a foot ulcer in a person with current or previously diagnosed diabetes mellitus, and usually accompanied by diabetic peripheral neuropathy (DPN) and/or diabetic peripheral artery disease (DPAD) in the lower extremity” [10].

DFUs are one of the most studied types of chronic wound, due to their public health importance and wide social impact [6]. As of 2021, approximately 10% of the global adult population, 536.6 million individuals, live with diabetes [11]. Of these, 19–34% may develop DFUs during their lifetime [12], with around 18.6 million patients affected annually [13]. DFUs have a prevalence of 6% and pose higher risks in patients with type II diabetes and in males [14]. A meta‐analysis estimated the prevalence of diabetic foot at risk of ulceration among patients with diabetes at 53.2% [15]. Alarmingly, half of these cases involve infections, making DFUs the leading cause of lower‐extremity amputations [16]. Infected foot ulcers carry a dire prognosis, demonstrating a 17% limb amputation rate and a 15% mortality rate within 1 year [17]. Healing rates are poor, with recurrence rates reported approximately 40% within 1 year and 65% within 5 years after healing [12]. In addition to their clinical burden, DFUs impose significant financial challenges, with annual medical cost, including assessment, nursing, and management, exceeding $35,000 per patient [18]. To prevent DFUs, patients with diabetes often face increased medication costs associated with managing comorbidities such as dyslipidemia and hypertension. A study conducted in China reported that patients with DPN, a major precursor to DFUs, had approximately 30% higher medication expenses compared with those without DPN [19]. These epidemiological figures emphasize the urgent need for effective strategies to mitigate the escalating medical and financial burden posed by DFUs on healthcare systems and affected families worldwide.

The clinical and economic challenges posed by DFUs underscore their public health importance. Despite decades of research, the pathogenesis of DFUs remains incompletely understood, and effective therapies are still lacking in clinical practice [20, 21, 22]. This review aims: (1) to synthesize current insights into the molecular mechanisms underlying DFUs, with particular focus on how metabolic dysregulation impairs wound healing, (2) to examine the critical preconditions that establish the foundation for DFU development, (3) to summarize the disruption of key cellular functions essential for wound repair within the DFU microenvironment, and (4) to critically evaluate animal models and emerging therapeutic strategies. Relevant studies were identified primarily via PubMed using keywords such as “diabetic foot ulcer” and “wound healing,” combined with terms like “neuropathy,” “vascular disease,” “metabolic disorder,” “macrophage,” “fibroblast,” “experimental model,” “exosome,” and “skin substitute,” with an emphasis on literature from the past 5 years.

## 2 Metabolic Dysregulation in Diabetes

DFUs occur in both type I and II diabetic patients. Type I diabetes is characterized by a chronic autoimmune destruction of pancreatic beta cells and a decrease in insulin production, while type II diabetes is characterized by insulin resistance and a progressive decline in the capacity of beta cells to produce insulin [23, 24]. Therefore, the core of the pathogenesis of diabetes and its complications lies in the insulin dysfunction. Insulin plays multiple crucial roles in the metabolism of carbohydrates and lipids. Cellular functions vary with the physiological and pathological alterations in metabolism. Thus, comprehensive knowledge of diabetes‐related metabolic disorder is vital for understanding the mechanism of DFUs.

Hyperglycemia is the hallmark in both types of diabetes and is the direct consequence of insulin dysfunction. Multiple metabolic pathways undergo alteration in response to the excessive glucose concentration, including the polyol pathway and hexosamine pathway, leading to the excessive production of reactive oxygen species (ROS) and advanced glycation end products (AGEs) [25]. The aberrant extracellular metabolic microenvironment and intracellular metabolites contribute to the dysregulation of signaling transduction and the worsening of insulin resistance [26, 27]. Moreover, hyperglycemia regulates chromatin histone modifications, DNA methyltransferase (DNMT) activity, noncoding RNA levels, and RNA modifications. These epigenetic mechanisms lead to the irreversible diabetic “metabolic memory” [28].

### 2.1 Glycolysis and Polyol Pathway

**FIGURE 1:**
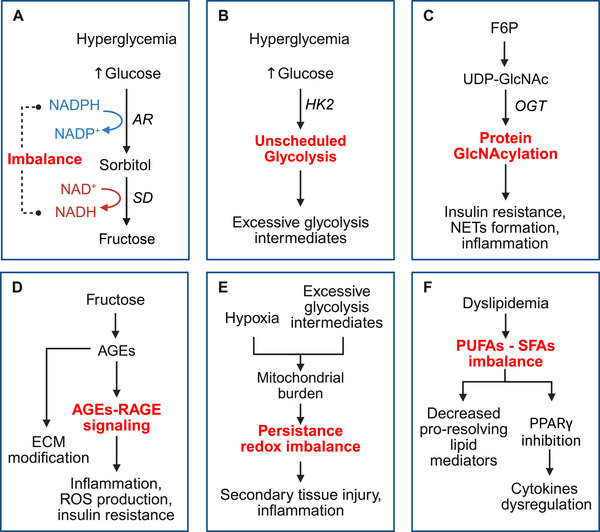
Metabolic dysregulation in diabetic cells. In diabetes, cellular metabolic abnormalities are driven by chronic hyperglycemia and insulin resistance, leading to impaired glucose uptake, mitochondrial dysfunction, and altered lipid metabolism. (A) The polyol pathway converts excess glucose to fructose via sequential catalysis by AR and SD, resulting in fructose accumulation, NADPH depletion, and NADH overproduction. (B) HK2‐mediated unscheduled glycolysis overload produces excess glycolysis intermediates, such as F6P. (C) F6P can be transformed into UDP‐GlcNAc and promote protein GlcNAcylation via OGT, disrupting insulin signaling and promoting inflammation. (D) Fructose‐derived AGEs engage RAGE receptors to activate proinflammatory signaling pathways and disrupt ECM structural integrity via glycation. (E) Hypoxia caused by low perfusion and excessive glycolysis intermediates increases mitochondrial burden, leading to ROS accumulation and reduced ATP production. (F) SFA accumulation and PUFA deficiency promote lipotoxicity and impair synthesis of proresolving lipid mediators. *Abbreviations*: AGEs, advanced glycation end products; OGT, O‐glycosyltransferase; ROS, reactive oxygen species; NADPH, nicotinamide adenine dinucleotide phosphate; NADH, nicotinamide adenine dinucleotide; F6P, fructose 6‐phosphate; HK, hexokinase; AR, aldose reductase; SD, sorbitol dehydrogenase; RAGE, receptor for AGEs; PUFA, polyunsaturated fatty acid; SFA, saturated fatty acid; ECM, extracellular matrix; PPARγ, peroxisome proliferator‐activated receptors gamma; NETs, neutrophil extracellular traps; UDP‐GlcNAc, uridine‐5′‐diphospho‐N‐acetylglucosamine. Created with BioRender.

Glycolysis, the initial step of glucose metabolism in the cytosol, converts glucose into pyruvate. Dysregulated glycolysis under diabetic conditions remodels multiple metabolic pathways. Under conditions of hyperglycemia, the first step of glycolysis, catalyzed by hexokinases (HKs), becomes saturated, resulting in the diversion of glucose from glycolysis to the polyol pathway [29]. The aldose reductase (AR) utilizes nicotinamide adenine dinucleotide phosphate (NADPH) to convert glucose into sorbitol, which is then converted to fructose by sorbitol dehydrogenase, generating nicotinamide adenine dinucleotide (NADH) [29]. Activation of the polyol pathway leads to excessive fructose accumulation and disrupts the NADPH/NADH balance (Figure 1A).

The polyol pathway has been implicated in the development of DFUs. Among the two major HK isozymes, HK‐2 participates in the enhanced unscheduled glycolysis observed in hyperglycemia. At high glucose concentrations, HK‐2 is protected from proteolysis, gaining extra stability and activity to produce excessive glucose‐6‐phosphate (G6P) without corresponding transcriptional or functional coordination [30] (Figure 1B). Elevated G6P levels displace HK‐2 from mitochondria, resulting in impaired adenosine triphosphate (ATP) disposal, mitochondrial membrane hyperpolarization, and increased ROS production [30]. Notably, HK‐2 has been identified as a hub mitophagy‐related gene in DFUs, with validated diagnostic potential [31]. In addition, through AR activation, hyperglycemia decreases the activity of RUNX family transcription factor 2 (RUNX2), a critical regulator of angiogenesis essential for wound repair [32]. Other studies have indicated that AR impairs diabetic wound healing and contributes to DPN, whereas AR‐targeted therapies can improve healing outcomes [33, 34].

### 2.2 Hexosamine Pathway and GlcNAcylation

The hexosamine pathway, which contributes to protein glycosylation, is implicated in the development of cellular insulin resistance. It utilizes fructose‐6‐phosphate, glucosamine (GlcN), or N‐acetylglucosamine (GlcNAc) to generate GlcNAc‐6‐phosphate and produce the substrate for O‐GlcNAcylation [35]. O‐GlcNAcylation can be found on functional and structural proteins at multiple sites within cells and plays crucial roles in the regulation of transcription, epigenetic modification and signaling dynamics (Figure 1C) [36]. A special case in metabolic dysregulation is the establishment of insulin resistance. Insulin induces the autophosphorylation of the receptors and the tyrosine phosphorylation of insulin receptor substrate 1 (IRS1) on tyrosine, which subsequently activates the phosphoinositide 3‐kinase (PI3K) to recruit and activate protein kinase B (AKT). The phosphorylation site of AKT can be masked by O‐GlcNAcylation, therefore hindering downstream signaling [37]. The extensive and excessive O‐GlcNAcylation results in dysregulation of gluconeogenesis, lipogenesis, and calcium homeostasis [35, 38].

Multiple studies have implicated elevated O‐GlcNAcylation in impaired diabetic wound healing. In keratinocytes, hyperglycemia‐driven increases in O‐GlcNAcylation reduce levels of galectin‐7, a factor that promotes wound healing [39]. Furthermore, O‐GlcNAcylation also contributes to the formation of neutrophil extracellular traps (NETs) in diabetic wounds and exacerbates fibroblast‐mediated inflammation [40] and their contraction [41]. Notably, inhibiting O‐glycosyltransferase promotes wound closure, highlighting O‐GlcNAcylation as a promising therapeutic target [42].

### 2.3 Formation and Effects of AGEs

Exogenous AGEs are abundant in modern western diet, which are one of the crucial reasons for obesity. Excessive glucose, fructose from polyol pathway, and glyceraldehyde derived from metabolic intermediates participate the production of endogenous AGEs. Highly reactive carbonyl precursors produced by Maillard reaction with the dicarbonyl stress induced by unscheduled glycolysis, polyol pathway and lipid peroxidation undergo rearrangement to become the AGEs [30, 43, 44]. AGEs are reported to exert effects either through direct cross‐linking with the proteins or via signal transduction mechanisms (Figure 1D). The AGEs activate several receptors, among which receptor for AGEs (RAGE) is mostly studied. RAGE is considered a type of pattern recognition receptor. The recognition of AGEs by RAGE activates several downstream effectors, including mitogen‐activated protein kinase (MAPK) family, Janus kinase family and protein kinase C (PKC), contributing to complex and extensive adverse effects on cellular function. PKC, extracellular regulated protein kinase, and c‐Jun N‐terminal kinase (JNK) are reported to mediate the phosphorylation of IRS1 on serine [27]. The phosphorylation of IRS1 on serine blocks its phosphorylation on tyrosine and inhibits the downstream insulin action [37].

The activation of multiple kinases also lead to the elevated activity of transcriptional regulators, such as nuclear factor kappa‐light‐chain‐enhancer of activated B (NF‐κB) and signal transducers and activators of transcription (STATs), and both contribute to the transcription of proinflammatory cytokines [43]. The activation of NF‐κB increases the transcription of inducible nitric oxide (NO) synthase to generate more ROS, while the latter in turn facilitates the production of AGEs [45]. This vicious cycle amplifies the ROS production, inflammatory response and insulin resistance. The accumulation of ROS and amplified inflammation critically impair diabetic wound healing. Moreover, the nonenzymatic modification of collagen fibers by AGEs contributes to the alteration in deposition, physical features, and structural integrity of extracellular matrix (ECM) [46]. Glycation alters both the mechanical properties of collagen matrices and their interactions with fibroblasts. In murine full‐thickness wound models, implantation of glycated collagen matrices significantly delays the healing process [47].

### 2.4 Accumulation of ROS

The glycolytic product pyruvate enters the mitochondria, where it is converted into acetyl‐CoA, which subsequently enters the tricarboxylic acid cycle. These processes generate NADH, which fuels the oxidative phosphorylation (OXPHOS) via the electron transport chain (ETC) located on the inner mitochondrial membrane. OXPHOS generates ATP and produces ROS as byproducts, which accumulate excessively under diabetic conditions (Figure 1E). ROS not only contribute to oxidative stress but also play a critical role in signal transduction [48]. Type II diabetes is characterized by mitochondrial dysfunction, high production of ROS and low production of ATP [49]. Mitochondrial dynamics, including biogenesis, mitophagy, fission, and fusion, were reported to play crucial roles in cellular functionality [50, 51, 52]. Excessive NADH from polyol pathway and unscheduled glycolysis aggravates the burden of complexes I, III, and IV, leading to the enhanced leak of electrons. This mediates one‐electron reduction of oxygen to superoxide, which is converted to hydrogen peroxide (H_2_O_2_) via dismutation [53]. Another contributor to ROS production is oxygen availability. Paradoxically, although ROS production requires oxygen, hypoxia enhances ROS generation. This increase may be attributed to the conformational changes in ETC complexes or the regulation from other extramitochondrial factors [48]. Glutathione (GSH) is oxidized by H_2_O_2_ into glutathione disulfide (GSSG) to alleviate oxidative stress. GSSG can then be reduced back to GSH in an NADPH‐dependent manner by GSH reductase [54, 55]. The consumption of NADPH by polyol pathway impairs antioxidant capacity and deteriorates the oxidative stress. ROS can also be generated from NADPH by NADPH oxidases (NOXs) during immune response, in which ROS plays an essential role in bacterial defense, inflammasome activation and innate immunity regulation [56]. The redox balance also plays significant role in proliferation, migration, motility and cell death [55, 57, 58]. The persistent accumulation of ROS in diabetic wounds is well established, and targeting ROS clearance is considered promising, both for direct therapeutic intervention and for the design of innovative wound dressing.

### 2.5 Dyslipidemia and Downstream Influence

Another significant metabolic alteration caused by insulin dysfunction is dyslipidemia. Despite its established association with obesity and type II diabetes, recent investigations indicate a strong correlation with type I diabetes as well [59, 60]. The chylomicrons bring lipids from small bowel postprandially into the circulation, where they are taken up by the liver. Liver produces very low density lipoproteins (LDL) (VLDLs) and converts them into LDLs by releasing the glycerol and fatty acids and leaving mainly cholesterol. LDLs circulate in blood and can be absorbed by cells with LDL receptors. On the one hand, insulin prevents dyslipidemia by decreasing the release of free fatty acids (FFAs) from adipose tissue through inhibiting hormone‐sensitive lipase and the production of VLDLs [59]. On the other hand, insulin induces FFAs uptake and lipogenesis by activating the sterol‐regulatory element binding protein pathway [37, 61]. In diabetes, insulin dysfunction results in the increased cholesterol and triacylglycerols. The increased FFAs in circulation also play roles in insulin resistance and participates in the vicious circle [60]. As the adipose tissue is crucial to lipid homeostasis and storage, adipocyte dysfunction results in adipocyte hypertrophy, excess collagen deposition, inflammatory response, and vascular remodeling in fat tissue. This dysfunction contributes to dyslipidemia and aberrant distribution and activity of adipose tissue [61]. Adipose tissue is dynamic in response to external stimuli. White adipose tissue (WAT) resides in the abdominal cavity surrounding major organs and blood vessels, as well as in subcutaneous depots, serving as a site for energy storage and endocrine regulation. Brown adipose tissue (BAT) represents less than 5% of adipose tissue and participates in thermogenesis and inflammation resolution [62]. The switch from WAT to BAT is called adipose tissue browning, whose dysregulation is of high correlation with insulin resistance [63, 64].

Lipid metabolism not only provides energy but also supplies ligands in signaling transduction as regulatory bioactive lipids. A wide variety of lipids have been shown to exhibit bioactive properties, including several glycerolipid‐derived molecules and sphingolipids [65]. These lipid mediators are mainly derived from fatty acids. There is an imbalance of saturated fatty acids (SFAs) and poly unsaturated fatty acids (PUFAs) in diabetic conditions, with SFAs mediating lipotoxicity in beta cells [66]. The western diet that contributes to obesity is rich in SFAs, while human lacks desaturase for the synthesis of PUFAs [66, 67]. This imbalance leads to an excessive inflammatory response as PUFAs are crucial ingredients for the production of proresolving lipid mediators (Figure 1F) [68]. During inflammation initiation, the ω‐6 PUFA, arachidonic acid is converted into a series of proinflammatory mediators to ignite the proinflammatory response. In phase of resolution, arachidonic acid is switched to produce anti‐inflammatory lipoxins and aspirin‐triggered lipoxins. The ω‐3 PUFAs also participate in generating several proresolving lipid mediators, including resolvins and protectins. Resolvins were also reported essential in NETs clearance by macrophage [69]. In diabetes with insulin dysfunction, the proinflammatory lipid mediators are upregulated and the intensity of the switch was decreased, contributing to chronic inflammation and also the insulin resistance [68].

To summarize, diabetic metabolic condition and insulin resistance mutually exacerbate each other. In this feedback circle, the excessive production of ROS, AGEs, and inflammation collectively drive diabetes‐related cellular dysfunction (Figure 1). Metabolic disorder not only lies at the core of diabetes but also holds the key to fully understanding the pathogenesis of its complications, including delayed wound healing and DFUs.

## 3 Predisposing Factors for Diabetic Wounds

**FIGURE 2:**
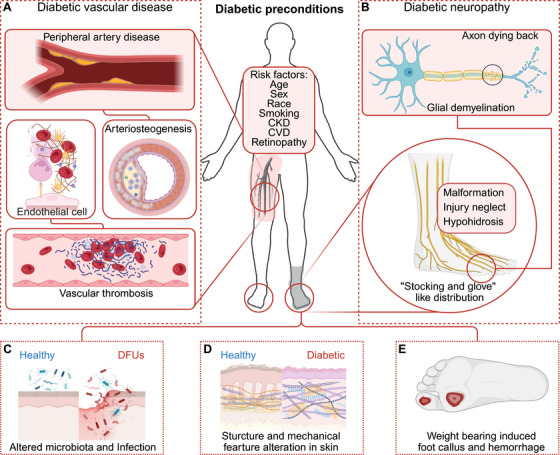
Contributing factors in diabetes leading to DFUs. Diabetes fosters a cascade of pathological preconditions that synergistically drive tissue damage and impaired healing. (A) Diabetic vascular complications cause chronic ischemia, limiting oxygen and nutrient delivery to tissues. (B) Diabetic neuropathy disrupts sensory, motor, and autonomic nerves, impairing injury detection, sweat gland function, and microvascular regulation. (C) Concurrently, dysregulated microbial colonization arises from hyperglycemia‐altered skin pH, immune dysfunction, and disrupted antimicrobial peptide secretion, promoting pathogenic overgrowth and biofilm formation. (D) Structural skin abnormalities, including epidermal thinning, collagen cross‐linking, and impaired barrier function, heighten susceptibility to mechanical stress and delayed repair. (E) Callus formation is resulted from alteration of skin structure and weight bearing, while minor trauma and inflammation can cause hemorrhage. Together, these factors create a high‐risk milieu for chronic wounds, infections, and diabetic foot ulcers. *Abbreviations*: DFUs, diabetic foot ulcers; CKD, chronic kidney disease; CVD, chronic venous disease. Created with BioRender.

The development of DFUs arises from a complex interplay of multiple pathological conditions. Among these, DPAD and DPN are the most recognized contributors. Based on etiology, DFUs can be categorized into three primary types: ischemia‐based, neuropathy‐based, and mixed type. Notably, the mixed type accounts for nearly half of all DFU cases, underscoring the critical roles that both vascular and neuropathic dysfunctions play in their pathogenesis [70]. Although peripheral ischemia has been studied in greater depth in the context of obesity and arteriosclerosis, DPN appears to play a more significant role in the recurrence of DFUs because the loss of sensation against noxious stimulation by profound neuropathy cannot be corrected by surgery [12]. Neuropathic ulcers are typically painless and present as round, “punched‐out” lesions on weight‐bearing areas of the foot, often surrounded by thickened calluses. In contrast, ischemic ulcers are characterized by irregularly shaped lesions with pale or necrotic bases. They are often larger than neuropathic ulcers and are associated with higher risks of complications, such as cellulitis, abscesses, or osteomyelitis. Severe cases of ischemic ulcers may progress to gangrene, further complicating the clinical scenario [70, 71]. Additionally, structural alterations in the skin and shifts in the microbiota community also contribute to DFU formation, potentially influencing inflammation and infection dynamics (Figure 2).

### 3.1 Diabetic Vascular Disease

Diabetes is associated with long‐term damage to both large and small, arterial and venous blood vessels throughout the body (Figure 2A). DPAD refers to narrowing or blockage of blood vessels in the limbs in diabetic condition, leading to ischemia mainly in lower extremities [72]. The classic symptom of peripheral artery disease is intermittent claudication, which might contribute to DPN in motor nerve to the abnormal gait. The endothelial dysfunction is recognized as one of major causes of diabetic vascular disease. It has been well described in the atherosclerosis and obesity. The excessive activation of MAPK pathway due to diabetic metabolic disorder results in dysregulated expression of vascular cell adhesion protein 1, intercellular adhesion molecule 1, E‐selectin, and plasminogen activator inhibitor‐1 (PAI‐1), dampening the endothelial function [73]. Elevated ROS damages the mitochondrial function and intracellular calcium homeostasis, results in the dysregulation of motility, proliferation, angiogenesis and increases DNA damage and apoptosis [74, 75]. Inflammatory signals are highly activated to promote the production of macrophage inflammatory protein 1, IL‐1β and TNF‐α [76]. Chemokines, such as CCL5 and CXCL10, are also upregulated and contribute to the recruitment of immune cells with the coordination of inflammatory cytokines [75]. Macrophages from the blood stream perform the efferocytosis and phagocytosis of apoptotic cells and the cholesterol‐rich lipoproteins accumulated in artery, transform into proinflammatory foamy‐like cells and gain the volume under the endangium, reducing the flow and the plasticity of the vessel in atherosclerotic cardiovascular disease [77].

However, DPAD is more likely to present as diffuse disease, characterized by long‐segment arterial occlusion and medial calcification in the layer composed of smooth muscle cells and the elastic membrane [70, 78]. The calcification props up the blood vessels from compression to conceal the artery dysfunction by unreliable ankle–brachial index, making DPAD often unnoticed in clinical screening [70, 79]. The initiation of microcalcification usually starts in the cholesterol‐enriched lipid pool, with the participation of vascular smooth muscle cells (VSMCs) and macrophages [78]. The process of vascular calcification is suggested to be a hydroxyapatite mineralization while the VSMCs may undergo osteogenic transformation, with diabetic specific effectors playing essential roles. RAGE–AGEs signaling downregulates the expression of markers in VSMCs and induces the activity of alkaline phosphatase and the transcriptional level of RUNX2, which are responsible for hydroxyapatite deposition, mineralization, and osteogenic differentiation [80, 81]. The downstream activation of MAPK pathway is responsible for the AGEs‐mediated phenotype transition [82, 83]. Another signaling cascade activated by AGEs is the transforming growth factor beta (TGF‐β) pathway, which activates the downstream SMAD family member 2 (SMAD2) to patriciate the type I collagen deposition and fibrosis [84]. Diabetic VSMCs express excessive S100 calcium binding protein A12 (S100A12), identified as a proinflammatory cytokine and a RAGE ligand, and contribute to the vascular calcification by NOX‐mediated oxidative stress [85]. PKC activation by AGEs participates in the transcriptional imbalance in genes involved in vascular dilation and contraction [78]. Macrophages may ingest the basic calcium phosphate and secret multiple cytokines including IL‐8 and TNFα, while the latter contribute to the calcification of VSMCs by suppressing the pyrophosphate [86]. Moreover, a considerable population of diabetic patients suffer from diabetic nephropathy, resulting in the dysregulation of serum phosphate and hormone involved in calcium and phosphate regulation. It has been recognized that there is high correlation between hyperphosphatemia and vascular calcification, contributing to the precondition of DPAD [87].

Apart from the arterial disorder, diabetes was considered as risk factors for venous thromboembolism (VTE) [88]. Despite controversial results in the association of VTE and type I or II diabetes, it has been recognized that hyperglycemia contributes to overall worse clinical outcomes with VTE in terms of mortality and long‐term complications in diabetes, due to the interplay between hyperglycemia, chronic inflammation, and the hypercoagulable conditions [89]. ROS and AGEs contribute to the release of NETs and NETosis [90]. On the base of endothelial dysfunction, NETs activate the thrombosis and play a role as the scaffold of fibrin formation [91]. The cascade of coagulation is energized with elevation of multiple blood coagulation factors, thrombin and fibrinogen. In addition to whose formation, thrombus in diabetic condition show greater resistance to the fibrinolytic system due to the elevated level of PAI‐1 [89]. Platelet hyperactivity can be induced by the activation of PKC pathway to trigger the platelet activation, while the increased glycation of platelet surface proteins reduces the fluidity of membrane and enhances the adhesion [92].

### 3.2 Diabetic Neuropathy

DPN is a length‐dependent neurodegenerative disorder of peripheral nerve system that preferentially targets sensory, autonomic and motor axons (Figure 2B). Progressive DPN involves retraction and “dying back” of axonal terminals while the proximal part remains less affected, causing the “stocking and glove like” distribution in extremities. Of note, DPN is also considered as a result of diabetic microvascular disease, apart from the predictable sufferings from adverse metabolic environment in diabetes. The most common structural abnormality in diabetic vasa nervorum, which supplies the nerve and axon, is the thickening of endoneurial basement membrane. Hyperglycemia and decreased NO level inhibit the vasodilation, with the hyperplasia and hypertrophy in endothelial cells in small endoneurial vessels, leading to hypoxia and neuronal damage [93, 94].

The dysregulation of both axon and glia contribute to the development of DPN, while the aberrant crosstalk between them also accelerate the pathogenesis. For the maintenance specialized functions, including releasing and recycling the neural transmitters and membrane potential activity, neuron demands greater energy provided by special axonal biogenesis and trafficking of mitochondria and the schwann–axon metabolic coupling [95]. In schwann cells, pyruvate generated from glycolysis is converted into lactate, while the latter is transferred into energy‐hungry axons via monocarboxylate transporters (MCTs), which are crucial for homeostasis of axon. MCT1 is decreased in diabetic peripheral nerve while the reduction of MCT1 leads to a worse impairment in sensory and motor conductions and severe nerve demyelination [96]. MCT1 was also reported to contribute to the maintenance of senescent nerve myelination and the motor end‐plate innervation in mice model [97, 98]. Schwann cells undergo glycolytic shift and produce more lactate by mammalian target of rapamycin (mTOR) activation, reducing the axonal degeneration in peripheral nerve injury [99]. However, excessive production of lactate in schwann cell specific Ras homolog mTORC1 binding knockout mice results in axonal oxidative stress and degeneration [100]. Ambivalent knowledge of lactate in peripheral nerve system demands further investigation and validation in the role of metabolic coupling in DPN.

With the vascular dysfunction in DPN, axons suffer from hypoxia and produce excessive ROS and less ATP, resulting in a scarce of energy in axon. Inhibited lactate shunt aggravates the bioenergetic failure. Mitochondria trafficking is inhibited by energy shortage since the mitochondrial kinesin motor also demands for ATP [101]. Milton, mitochondrial motor‐adaptor protein is modified by O‐GlcNAcylation to inhibit the trafficking in hyperglycemia [102]. Furthermore, the genes related to mitochondrial biogenesis and OXPHOS are also downregulated in type II diabetes [49]. With the process of axon degradation, serum neurofilament (NF) light chain level is considered as the biomarker of early diabetic sensorimotor polyneuropathy [103]. NFs are cylindrical cytoskeleton present in myelinated axons of the neurons, maintaining the shapes and providing structural support. As the basis of DPN, the decrease of NFs roots in the posttranscriptional degradation led by oxidative stress or glycation and the transcriptional inhibition with decreased mRNA level [104].

Schwann cell and axon can utilize lipids as the source of energy by β‐oxidation, while schwann cell also take in fatty acids for the generation of myelin. Diabetic dyslipidemia contributes to the DPN by depolarizing the mitochondria, disturbing the mitochondrial dynamics and producing excessive ROS by lipid peroxidation [105]. Severely mitochondrial dysfunction with respiration uncoupled from ATP production has been observed in animal with western diet [106]. Excessive SFAs are converted into lipotoxic acylcarnitine in schwann cells, which may be transferred into axon [105].

This combination of reduced energy production, increased ROS, degraded axonal structure leads to the dysregulation in feelings, motion and autonomic activity. Sensory neuropathy leads to loss of proprioception, pain, or temperature sensation, resulting in the deficiency of in‐time recognition of minor injury. The motor neuropathy reduced the muscle domination and induced muscle waste. Both motor and sensory neuropathy contribute to the deformed gait and malformation. With the excessive weight bearing, the abnormal distribution of pressure on pelma caused the callus and hemorrhage beneath it. The removal of callus reveals the ulcer through skin and the subcutaneous structure. Sympathetic autonomic dysfunction decreases the sweating and causes skin dryness and cracking, contributing to the fragility of barrier [16].

### 3.3 Bacterial Colonization

The microbiota plays a crucial role in skin defense and homeostasis. In a healthy state, skin commensal microbes coexist harmoniously with their human hosts, preventing the colonization of potential pathogens. However, in individuals with impaired immune responses and a dysregulated microenvironment, such as those with diabetes, these protective mechanisms can be compromised, allowing pathogenic bacteria to colonize wounded tissue [2] (Figure 2C). Studies have shown differences in the bacterial communities of diabetic skin versus healthy skin, as well as DFUs versus adjacent healthy skin [107, 108]. Diabetic skin typically has a reduced bacterial diversity and colony compared with healthy skin [109]. The genus *Chryseobacterium* and *Lactobacillaceae Roseomonas* are more abundant in healthy skin, whereas *Prevotella, Vagococcus, Peptostreptococcus*, and *Leptotrichiaceae* are prevalent in DFU skin [109, 110].

The discrepancies in findings across studies can be attributed to differences in study design, sampling sites, and patient heterogeneity. Notably, the crosstalk between the microbiota and the immune system, as well as interactions between different bacterial colonies, can influence healing outcomes. For example, the pathogenic effects of anaerobes may be enhanced by aerobes, which consume oxygen and promote anaerobic growth. This crosstalk can contribute to the formation of polymicrobial biofilms, which are associated with adverse outcomes in DFUs [111].

### 3.4 Alteration of Skin Architecture

While the roles of diabetic vascular disease, neuropathy, and infection in the development of DFUs have been well recognized, alterations in the characteristics and structure of diabetic skin warrant greater attention (Figure 2D). Skin can be conceptualized as a multilayered composite material, and its structural vulnerabilities may represent a primary defect contributing to DFU formation. However, investigations into these structural changes remain limited.

A study using a diabetic rat model demonstrated a negative correlation between glycosylated hemoglobin and collagen areal density, along with a positive correlation with orientational complexities of collagen, indicating that excessive glycosylation adversely impacts ECM behavior. In diabetic skin, the epidermis is thinner than in healthy controls, and the epidermis–dermis junction is reduced, compromising structural integrity [112]. These findings align with observations in diabetic patients, where alterations in the epidermis–dermis junction are associated with reduced skin elasticity [113]. Additionally, research has shown that diabetes and hypercholesterolemia impair epidermal cornification during fetal skin development in rats [114]. In a streptozotocin‐induced diabetic mouse model mimicking type 1 diabetes, cell density is decreased in both epidermis and dermis [115].

Structural changes extend beyond the skin to deeper tissues, including the plantar fascia. Diabetic individuals exhibit a thicker and stiffer plantar fascia, accompanied by smaller fat pads [116]. In diabetic septa, collagen and elastic fibers appear fragmented and irregularly deposited, impairing their ability to support and distribute forces in the subcutaneous layer [116]. These changes are likely driven by AGEs, which interfere with ECM integrity and regulate mechanical properties at various hierarchical levels of collagen in tendons [116]. We previously reported that the movement of fascia tissue plays a crucial role in wound bed padding and scar formation [117]. In diabetes, the stiff and dysfunctional fascia may hinder the initiation of wound repair, exacerbating poor healing outcomes. Additionally, diabetic skin contains a higher proportion of immature collagen, further contributing to its compromised structural and functional integrity [115].

In summary, the structural and functional alterations in diabetic skin, vasculature, and nervous system create a fragile skin barrier with limited healing capacity. The drying and thinning of the diabetic epidermis leave it vulnerable to minor injuries, which often go unnoticed due to reduced sensation. Impaired blood supply leads to localized hypoxia and increased susceptibility to infection, further complicating ulcer management. Additionally, motor abnormalities and obesity contribute to concentrated pressure on specific areas of the foot, resulting in callus formation and heightened risk of ulceration (Figure 2E). These interconnected preconditions emphasize the multifaceted challenges in managing DFUs. Addressing each of these aspects is crucial for effective clinical strategies to improve outcomes in diabetic wound care.

## 4 Cellular Mechanisms of Impaired Wound Healing in Diabetes

To understand the mechanisms underlying delayed wound healing in diabetes, it is crucial to first outline the physiological processes involved in normal wound repair. Under normal conditions, wound healing proceeds through four overlapping but sequential phases: hemostasis, inflammation, proliferation, and tissue remodeling [8]. Each phase requires the precise coordination of cellular activities, including activation, migration, proliferation, ECM deposition, and remodeling. These events are further orchestrated by a tightly regulated inflammatory response and sufficient angiogenesis [1, 5]. Any disruption in the sequence, intensity, or duration of these processes can result in delayed or impaired healing, often manifesting as chronic wounds or excessive scar formation. A hallmark of chronic wound healing, such as diabetic wounds, is the asynchronous progression of healing phases. This is typically characterized by prolonged and excessive inflammation, insufficient angiogenesis, and impaired re‐epithelialization [6]. In this section, we focus on the behavior of key cellular players during wound healing and discuss the influential factors that disrupt their normal function in the diabetic context. By comparing cellular responses in healthy and diabetic conditions, we aim to shed light on the mechanisms contributing to delayed healing in diabetes.

### 4.1 Platelets and Coagulation

Platelets serve as the “first responders” in wound healing and immune responses [118]. Immediately following injury, blood vessels constrict to minimize blood loss, aided by clot formation. Platelets are the first to arrive at the site of acute injury, where they are activated upon contact with the exposed subendothelial matrix and collagen, initiating the coagulation cascade [119]. Once the platelet plug is formed, activated platelets release bioactive molecules stored in their granules, which amplify coagulation, seal the exposed tissue, and recruit immune cells to the wound site through chemokine release and direct capture from the bloodstream [119, 120]. P‐selectin, translocated from platelet granules to the cell surface, facilitates the recruitment of monocytes, neutrophils, and lymphocytes, initiating the inflammatory phase and supporting neutrophil transendothelial migration [121]. In addition, platelets release multiple growth factors, such as TGF‐β, platelet‐derived growth factors (PDGF), and vascular endothelial growth factors (VEGF), which are essential for activating, migrating, and proliferating tissue repair cells [119, 122]. Platelets also participate in antimicrobial defense through direct interaction with bacteria via surface receptors like TLR2 and TLR4 or indirect interaction through plasma proteins. This positions them as a critical line of defense against wound infection [123]. Notably, platelet lysates have been shown to inhibit bacterial growth while promoting re‐epithelialization and granulation tissue formation, particularly in burn wounds with multidrug‐resistant infections [124].

Although limited studies have explored platelet dysfunction specifically in diabetic wound healing or DFUs, their role in clinical outcomes is gaining interest. Platelet‐related parameters, such as the platelet‐to‐lymphocyte ratio, have been associated with DFU outcomes, including morbidity, mortality, and the risks of osteomyelitis and amputation [125, 126, 127, 128]. A higher platelet‐to‐lymphocyte ratio generally predicts worse outcomes and correlates with increased inpatient care costs [129]. However, findings regarding platelet counts alone as prognostic indicators in DFUs remain inconsistent and controversial [130, 131]. Despite the intricate relationship between hypercoagulability and platelet function in diabetes, platelet‐based therapies have demonstrated promise for chronic wound healing. Both animal studies and clinical trials highlight the benefits of topical applications of platelet‐enriched lysates or platelet‐derived factors, which enhance various aspects of diabetic wound healing [132, 133, 134].

### 4.2 Neutrophils

Neutrophils are the first immune cells recruited to the wound site, playing a critical role in the early inflammatory phase of wound healing [135]. Their rapid accumulation and timely resolution are essential for preventing chronic inflammation [1]. Upon injury, neutrophils sense recruiting signals such as damage‐associated molecular patterns (DAMPs) and pathogen‐associated molecular patterns (PAMPs), enabling their adhesion to activated endothelial cells expressing selectins [1, 136, 137, 138]. Once activated, neutrophils initiate a leukotriene B4‐dependent positive feedback loop that drives their collective migration to the injury site, namely swarming behavior, leading to further recruitment and localized amplification of inflammation [139].

**FIGURE 3:**
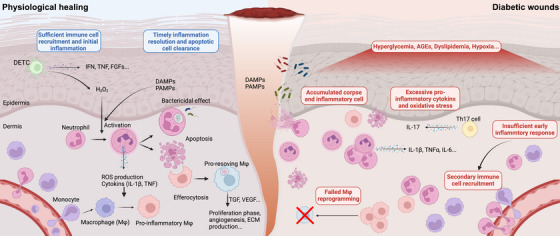
Immune response in physiological wounds versus diabetic wounds. Diabetic wounds are defined by profound immune dysregulation, disrupting the tightly orchestrated healing cascade. Under physiological conditions (left panel), neutrophils rapidly infiltrate wounds within 24 h to eliminate pathogens via phagocytosis and ROS production. Subsequently, macrophages migrate to the site, and undergo efferocytosis to clear the cellular corpse. In diabetic wounds (right panel), however, disordered metabolic microenvironment perturbs these dynamics: neutrophils produce excessive ROS and transform into NETosis, while macrophages display impaired efferocytic capacity and remain “locked” in a proinflammatory state. This functional chaos fosters a self‐amplifying cycle of oxidative stress and chronic inflammation. Secondary immune cell infiltration further exacerbates tissue damage and stalls wound closure. *Abbreviations*: DAMPs, damage associated molecular patterns; PAMPs, pathogen‐associated molecular patterns; DETC, dendritic epidermal T cell; Th17, T helper cell 17; Mφ, macrophage; AGEs, advanced glycation end products; TNF, tumor necrosis factor; TGF, transforming growth factor; VEGF, vascular endothelial growth factor. Created with BioRender.

Neutrophils contribute to pathogen defense and tissue repair by releasing proinflammatory cytokines (e.g., IL‐1β, IL‐6, and TNFα), ROS, and NETs [140] (Figure 3, left panel). NETs, composed of cell‐free DNA, antimicrobial peptides (AMPs), citrullinated histones, and activated proteins, effectively trap and eliminate pathogens [141]. However, excessive NET activation can damage surrounding tissues, impair vasculature, and exacerbate inflammation [142, 143]. To prevent such damage, neutrophils employ G‐protein‐coupled receptors‐mediated negative feedback loops to limit recruitment, and excessive NETs are cleared by macrophage‐mediated phagocytosis [144, 145]. Additionally, neutrophil swarming is implicated in ECM transport in surface injuries, suggesting a potential role in subcutaneous fascia movement into wound beds [146].

In diabetic wounds, neutrophil dysfunction contributes to impaired healing. Recruitment defects have been linked to dysregulation of forkhead box M1 and impaired formyl peptide receptor signaling [147, 148]. Reduced PAMP release from diabetic skin microbiota, such as *Pseudomonas aeruginosa*, further limits neutrophil attraction. Interestingly, exogenous lipopolysaccharide stimulation in the early phase can enhance neutrophil recruitment and accelerate diabetic wound healing [149].

NETs are enriched in nonhealing DFUs, where elevated neutrophil elastase levels are associated with infection and worsened ulcer progression [150]. Excessive NETs impair angiogenesis by inhibiting the Hippo–Yes‐associated protein (YAP)–SMAD2 pathway, and their formation promotes inflammasome activation and IL‐1β release. Genetic or pharmacological inhibition of NET formation accelerates wound healing in diabetic rodent models by reducing excessive inflammation [151, 152, 153, 154, 155]. Moreover, direct depletion of neutrophils has been shown to accelerate sterile wound healing, highlighting the importance of controlled neutrophil activity [156]. The seemingly contradictory roles of neutrophils stem from the dysregulated timing of immune responses in diabetic wounds. Both timely neutrophil infiltration and subsequent clearance are crucial for proper wound repair. Failure to achieve this balance in diabetes disrupts immune coordination, prolongs inflammation, and delays healing (Figure 3, right panel).

### 4.3 Macrophages

Macrophages, derived from infiltrating monocytes, play a pivotal role in wound healing by orchestrating inflammation and tissue repair. These cells exhibit remarkable phenotypic plasticity, transitioning between proinflammatory (M1) and proresolving (M2) states depending on environmental cues. M1 macrophages dominate during the inflammatory phase, producing cytokines like IL‐1β, TNFα, and IL‐6, as well as matrix metalloproteinases (MMPs), which collectively combat pathogens and clear cellular debris. In contrast, M2 macrophages emerge during the later phases to promote tissue repair and remodeling, characterized by the production of arginase, TGF‐β, IL‐10, CCL18, and insulin‐like growth factors [157].

Although traditionally viewed as beneficial (M2) or detrimental (M1), recent studies have challenged this oversimplification of their phenotypes. Single‐cell analysis of DFUs revealed a paradox: healing wounds exhibit more “M1‐like” macrophages, whereas nonhealing wounds are dominated by “M2‐like” macrophages. This suggests that a failure to resolve inflammation, rather than macrophage polarity alone, underlies impaired healing in diabetes [147, 158, 159, 160]. In particular, macrophage phenotypic reprogramming in mouse models, from proinflammatory (F4/80^+^Ly6C^high^) to proresolving (F4/80^+^Ly6C^low^), is a key event marking the transition from inflammation to tissue repair. Prolonged retention of inflammatory macrophages and defects in this transition are hallmarks of diabetic wounds [161, 162, 163]. Macrophage efferocytosis, the process by which macrophages clear apoptotic neutrophils, is crucial for resolving inflammation and initiating tissue repair [164, 165]. Efficient efferocytosis not only prevents secondary necrosis of dying cells but also provides metabolic cues for macrophage reprogramming [164, 166] (Figure 3, left panel). Metabolites from apoptotic cells, such as sterols, amino acids, and nucleotides, activate lipid‐sensing pathways, including liver‐X receptors and peroxisome proliferator‐activated receptor gamma, to suppress inflammatory gene expression [165, 167]. Additionally, oxysterols derived from apoptotic cells inhibit the NLRP3 inflammasome, further limiting inflammation [168]. However, impaired efferocytosis is widely reported in diabetes, contributing to persistent inflammation and delayed healing.

Multiple mechanisms underpin defective efferocytosis in diabetic wounds. Decreased levels of bridging molecules such as milk fat globule‐epidermal growth factor (EGF) factor 8 (MFG‐E8) [169] and growth arrest‐specific 6 (GAS6) [170] impair apoptotic cell binding and uptake. For example, MFG‐E8 deficiency in diabetic patients and rodent models leads to apoptotic cell accumulation and prolonged inflammation, while MFG‐E8 supplementation improves wound healing [155, 169]. Similarly, defects in triggering receptor expressed on myeloid cells 2 (TREM2)‐dependent efferocytosis in obesity further highlight the metabolic links to impaired macrophage function [171].

NLRP3 inflammasome hyperactivation during macrophage efferocytosis is another key contributor to delayed healing in diabetic wounds. Dyslipidemia, common in diabetes, enhances macrophage lipid uptake via CD36, stabilizing NLRP3 and promoting excessive IL‐1β production [172, 173]. SFAs and intracellular crystallization further activate NLRP3, leading to lysosomal instability and inflammasome assembly [174]. Additionally, excessive NETs aggravate NLRP3 activation, which can be alleviated by DNase I treatment, demonstrating a link between neutrophil activity and macrophage dysfunction [154].

Together, these findings reveal that impaired macrophage phenotypic transition, defective efferocytosis, and excessive inflammasome activation synergistically contribute to delayed wound healing in diabetes (Figure 3, right panel). Targeting macrophage reprogramming, restoring efferocytosis, and mitigating NLRP3 activation show significant therapeutic potential for improving diabetic wound outcomes.

### 4.4 Other Immune Cells

In addition to macrophages and neutrophils, other immune cells also play significant roles in skin homeostasis and repair. Dendritic cells (DCs) are a diverse population crucial for bridging innate and adaptive immune responses. These cells capture, process, and present antigens, which are essential for T cell differentiation and activation. Langerhans cells, a subset of DCs residing in the epidermis, are particularly important in local immune responses [175]. DCs also interact closely with the nervous system to maintain skin homeostasis [176]. Prolonged hyperglycemia induces DC apoptosis and reduces both DC density and associated sensory nerve endings in diabetic wounds [177]. Interestingly, genetic manipulation of N‐myc downregulated gene 2 preserves the immature phenotype of DCs and promotes diabetic wound healing, while increased levels of solute carrier family 7 member 11 impair DC efferocytosis in this setting [178].

Skin harbors γδ T cells and regulatory T (Treg) cells, both of which support wound repair and immune regulation [179]. In human skin, γδ T cells represent a minor fraction of the total T cell population, accounting for approximately 2–9% in the dermis and 1–10% in the epidermis, while murine skin exhibits a unique dominance of epidermal γδ T cells, where the canonical Vγ5⁺ dendritic epidermal T‐cell subset (DETCs) comprises over 90% of epidermal T cells. This striking interspecies disparity underscores the distinct immunological roles and organizational principles of γδ T cells in skin homeostasis and disease models between humans and mice [180]. DETCs are reported to respond to injuries by recruiting macrophages and producing growth factors critical for repair [181, 182]. In diabetic wounds, γδ T cells exhibit reduced population size and delayed activation due to hyperglycemia [183], while Treg cells accumulate and exert beneficial effects on healing [184]. Dermal γδ T cells produce IL‐17, which inhibits IGF‐1 production by DETCs, whereas Treg cells counteract IL‐17‐driven inflammation [185, 186]. Elevated IL‐17 levels in diabetic wounds correlate with delayed healing, and blocking this pathway can improve outcomes [187, 188] (Figure 3, right panel).

Mast cells, another key immune cell type, secrete a wide array of bioactive factors, including growth factors and inflammatory cytokines, and play dual roles in wound healing. On one hand, mast cell degranulation has been found to increase in diabetic skin and rodent wound models, with stabilizers enhancing repair. On the other hand, wound healing is impaired in mice lacking mast cells, highlighting their essential role in tissue repair [189]. A hallmark of diabetic wound healing is the prolonged, dysregulated immune response characterized by chronic inflammation and aberrant cellular signaling (Figure 3). This pathology stems from multifactorial dysregulation—including impaired immune cell crosstalk, metabolic dysfunction, and unresolved oxidative stress—which disrupts the transition from inflammation to proliferation, perpetuating a nonhealing state. Restoring immune homeostasis through targeted modulation of these pathways represents a critical therapeutic strategy to reestablish physiological wound repair in diabetes.

### 4.5 Keratinocytes and Re‐Epithelialization

Keratinocyte proliferation and re‐epithelialization are critical for restoring the skin barrier function following injury. However, this process is significantly impaired in diabetic wound repair.

In addition to their role in tissue repair, keratinocytes serve as the first line of defense against pathogen invasion. In response to a nonsterile environment, keratinocytes produce antimicrobial bioactive factors, such as β‐defensins and cathelicidin (LL‐37), to protect against infection [190, 191]. LL‐37 is markedly upregulated at the margins of acute human surgical wounds but is notably absent at the edges of chronic, nonhealing wounds. Blocking LL‐37 function with antibodies hampers wound epithelialization [192]. In DFUs, LL‐37 levels are reduced compared with healthy skin, and a recent clinical trial demonstrated that topical LL‐37 cream enhances healing rates in DFUs with mild infections [193, 194].

As the outermost barrier, keratinocytes are the first to experience external forces during injury. In response, they release DAMPs, which together with PAMPs, activate sentinel cells in the skin and bloodstream [6]. Keratinocytes also propagate direct injury signals such as calcium ion (Ca^2+^) waves and ROS. Ca^2+^ waves, resulting from ion leaks due to membrane damage, spread through gap junctions or phospholipase C signaling pathways [195]. These signals induce H_2_O_2_ production, which further recruits inflammatory cells to the wound [196].

Keratinocytes integrate external signals and endogenous factors to promote healing. For example, H_2_O_2_ activates the MAPK pathway, stimulating keratinocyte proliferation within minutes of injury to initiate re‐epithelialization [197]. Growth factors such as fibroblast growth factors (FGF)‐10 also enhance keratinocyte migration and proliferation, critical steps in wound closure [198]. However, most of these growth factors are significantly reduced in diabetic wounds, limiting keratinocyte response.

Although keratinocyte proliferation begins shortly after injury, successful re‐epithelialization depends on ECM deposition and granulation tissue formation, which provide the structural support necessary for keratinocyte migration and adhesion [199]. Both processes are severely impaired in diabetic wounds, resulting in delayed and incomplete re‐epithelialization.

### 4.6 Fibroblasts and ECM

Fibroblasts are the principal cellular component of connective tissues and play essential roles in producing and remodeling the ECM. These cells are highly heterogeneous, exhibiting dynamic phenotypes and diverse functions during skin development, wound repair, and scar formation [200]. This heterogeneity arises from differences in lineage origin, anatomical location, and skin depth.

Based on the skin depth, fibroblasts can be classified into dermal papilla fibroblasts, papillary dermal fibroblasts, reticular dermal fibroblasts, and hypodermal fascia fibroblasts [201]. Among them, fascia‐resident fibroblasts have recently been recognized as a key player in wound healing and scarring [117]. Lineage‐based classifications further reveal functional diversity. For instance, CD26^+^Sca1^−^Dlk1^−^ fibroblasts contribute to the papillary dermis, while CD26^−^Sca1^+^ fibroblasts populate the reticular dermis and hypodermis. Dermal papillae cells, identified by Sox2 expression, may arise from papillary fibroblasts [201]. In murine back skin, two major fibroblast lineages, distinguished by the history of engrailed‐1 (En1) expression during early development, are crucial for wound healing outcomes. En1‐lineage naive fibroblasts dominate early in skin development but are later replaced by En1‐lineage past fibroblasts (EPFs), which drive wound contraction and ECM remodeling during repair [202]. CD26 serves as a key surface marker for EPFs [203]. Although fibroblast lineage dynamics are well characterized in physiological wound repair and scar formation, their roles in chronic wounds, such as diabetic ulcers, remain poorly understood.

**FIGURE 4:**
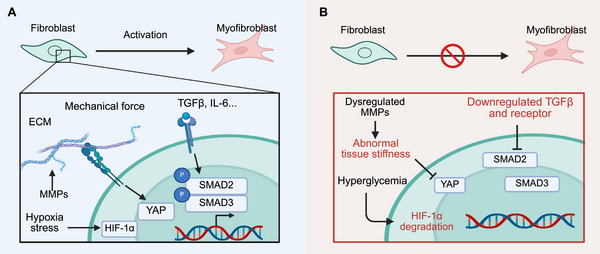
Fibroblast dysfunction in diabetic wound healing. (A) Fibroblast activation into myofibroblasts is essential for wound repair, driven by TGF‐β/IL‐6 signaling, hypoxia‐induced HIF‐1α, and YAP‐mediated mechanotransduction. (B) In diabetes, these pathways are disrupted by hyperglycemia, impaired cytokine signaling, and altered ECM mechanics, leading to defective myofibroblast activation and chronic wound formation. *Abbreviations*: ECM, extracellular matrix; MMP, matrix metalloproteinase; HIF‐1, hypoxia inducible factor‐1; YAP, Yes‐associated protein; TGF, transforming growth factor. Created with BioRender.

A critical event in wound healing is the transition of fibroblasts into contractile α‐SMA^+^ myofibroblasts, a process commonly referred to as fibroblast activation [204]. Myofibroblasts drive wound contraction and ECM deposition. However, this transition is impaired in chronic wounds, resulting in stalled repair [205]. Multiple pathways regulate fibroblast activation in normal wound healing, and dysfunction in each of these pathways has been implicated in diabetic wound pathology (Figure 4).

#### 4.6.1 Growth Factors and Inflammatory Cytokines

TGF‐β is the most well‐studied inducer of fibroblast activation, primarily signaling through the canonical SMAD2/3 pathways [206, 207], as well as through noncanonical routes including MAPK, NF‐κB, AKT, and ROCK [208]. In diabetic wounds, both TGF‐β levels and receptor expression are reduced [115]. Meanwhile, inflammatory NF‐κB signaling inhibits TGF‐β–SMAD pathway [115, 209, 210, 211]. Conversely, boosting SMAD activation accelerates diabetic wound repair [212, 213].

IL‐6 is another potent regulator that activates fibroblasts via STAT3‐dependent signaling to promote collagen synthesis [206, 214, 215]. It also indirectly amplifies this effect by inducing TGF‐β [216]. Notably, fibroblasts from diabetic wounds displayed reduced IL‐6 production and impaired responsiveness to IL‐6‐mediated migration in a mouse model [217]. Interestingly, scRNA‐seq analyses of human DFUs identified a cluster of prohealing fibroblasts that are enriched in healing compared with nonhealing wounds, characterized by elevated IL‐6 expression [158].

#### 4.6.2 Hypoxia and HIF‐1α Signaling

HIF‐1α is a master regulator of hypoxic response and plays a critical role in fibroblast‐mediated wound healing. Fibroblast‐specific deletion of HIF‐1α impairs both angiogenesis and ECM remodeling [218, 219, 220]. Recent findings demonstrate that HIF‐1α drives fibroblast transition from a PDPN^+^ proinflammatory state to a pSTAT3^+^ proto‐myofibroblast state, thereby facilitating SAMD2 activation downstream of TGF‐β in fascia fibroblasts. Loss of HIF‐1α impairs α‐SMA^+^ myofibroblast activation and skin contraction [221]. In diabetic wounds, HIF‐1α levels are notably reduced due to hyperglycemia‐driven proteasomal degradation [222, 223], and restoring its levels, for example, via hyperbaric oxygen therapy, improves healing outcomes [219, 224]. Human DFU studies further show that HIF‐1α‐high fibroblasts are enriched in healing wounds and may drive IL‐6 expression through transcriptional activation [158, 225].

#### 4.6.3 ECM Remodeling and MMPs

Activated fibroblasts also contribute to ECM remodeling, a process mediated by MMPs and their inhibitors (TIMPs). Prohealing fibroblasts in healing DFUs express elevated levels of MMP1, MMP3, MMP13, and TIMP1 [158]. While collagenases like MMP1 and MMP8 facilitate collagen turnover and wound resolution, gelatinases such as MMP2 and MMP9 are often detrimental, degrading essential factors like VEGF and dermatopontin [226, 227]. MMP activity is highly context‐dependent: their timing, substrate specificity, and regulation are crucial for physiological healing [226, 228, 229]. Proper MMP activity can promote fibroblast migration, but persistent overexpression of MMPs in diabetic wounds leads to excessive ECM degradation, impaired fibroblast activation, and defective remodeling [230].

#### 4.6.4 Mechanical Stress and YAP Signaling

Fibroblasts are mechanoresponsive cells that sense tissue stiffness and mechanical tension. YAP is a key transducer of mechanical signals and promotes fibroblast activation after injury [231]. Profibrotic EPFs, in particular, require YAP signaling for activation. In diabetic wounds, YAP is suppressed, impairing fibroblast function [232, 233]. Therapeutic modulations of YAP, for instance via enhancing ECM mechanotransduction with factors like agrin [234], simulation of ECM–cell interaction [235], or YAP‐targeting hydrogels [236], have shown promise for restoring fibroblast activation and promoting wound closure.

#### 4.6.5 Fibroblast Metabolism

Fibroblast metabolism undergoes significant reprogramming during wound healing, which is disrupted in diabetes. Pyruvate dehydrogenase kinase 4 (PDK4), normally induced by hypoxia to regulate mitochondrial metabolism [237, 238], is markedly downregulated in diabetic wounds. Restoring PDK4 expression rescues healing by sustaining HIF‐1α and YAP signaling [219, 239]. Additionally, rapid phosphorylation of ribosomal protein S6 (p‐RPS6) after injury marks metabolically active fibroblasts [240]; this signal is lost in diabetic wounds and correlates with poor repair [240, 241]. Accumulation of metabolic insults, including AGEs and their precursor 3‐deoxyglucosone (3DG), further impairs fibroblast adhesion and migration via dysregulation of MAPK signaling [242]. Hyperglycemia also reduces exendin‐4 level, a critical regulator of glycosaminoglycan (GAGs) synthesis, leading to defective ECM production and remodeling [243].

Collectively, fibroblasts are central to wound healing and scar formation, but their activation and function are severely compromised in diabetic wounds. Effective therapeutic strategies for diabetic ulcers will require restoring not just fibroblast activity, but also the supportive biochemical and biomechanical environment that enables their prorepair functions (Figure 4).

### 4.7 Endothelial Cells and Angiogenesis

Skin injury damages local vasculature, which must be repaired and significantly expanded to meet the increased oxygen and metabolic demands of the healing process. The dense network of vessels formed during repair gives granulation tissue its characteristic appearance and name. The angiogenic response begins within hours of injury, with sprouting vessel tips creating a capillary bed much denser than the surrounding normal tissue [1]. Endothelial cells are key players in this process, responding to proangiogenic factors such as VEGF, FGFs, and pigment epithelium‐derived factor to proliferate and elongate vessels [244, 245, 246]. Once vessels form, macrophages and pericytes support vascular maturation and integrity through factors like amphiregulin and TGF‐β [247].

In diabetic wounds, angiogenesis is severely impaired due to hypoxia, poor perfusion, and reduced levels of angiogenic factors. Additionally, abnormal ECM scaffold assembly disrupts the structural foundation for new vessel formation. Hyperglycemia exacerbates this dysfunction by increasing IL‐7 secretion from fibroblasts, which inhibits angiogenesis [248]. Furthermore, hyperglycemia enhances arginase activity in endothelial cells, depleting arginine, the precursor of NO, and impairing endothelial NO synthase activity. This leads to decreased NO production and increased superoxide anion generation, further impairing vascular function [249]. NO plays critical roles in tube formation, inflammation regulation, and antimicrobial defense. Hypoxia and NO deficiency together contribute to increased infection risk and biofilm formation in diabetic wounds. These findings highlight the therapeutic potential of NO and oxygen supplementation in promoting angiogenesis and improving diabetic wound healing outcomes [250].

### 4.8 Adipocytes

Recent research has uncovered the direct involvement of adipocytes and their precursors, preadipocytes, in wound healing. These cells contribute to antimicrobial defense, immune regulation, and fibroblast function, all of which are disrupted in diabetic wounds. In *Drosophila melanogaster* pupae, fat body cells (analogous to vertebrate adipocytes) migrate toward the wound site, sealing it, phagocytosing debris, and producing AMPs [251]. Vertebrate adipocytes likely share similar roles, as impaired adipogenesis in mice increases susceptibility to skin infections [252]. At the wound edge, adipocytes exhibit plasticity, transitioning to a fibroblastic morphology and secreting cathelicidin‐related AMP (CRAMP), a murine ortholog of human LL‐37, to exert antimicrobial effects [253].

In addition to their antimicrobial activity, adipocytes influence immune cell behavior, particularly macrophages and other myeloid cells. Following skin injury, adipocytes undergo lipolysis, releasing fatty acids that modulate macrophage immune signaling [254]. Fatty acid metabolism is often dysregulated in obesity and diabetes, leading to impaired immune responses [255, 256]. Deletion of adipocytes or inhibition of lipolysis beneath the skin delays wound healing and disrupts macrophage phenotype switching, further emphasizing their role in immune regulation [254]. Reduced lipolysis during the early inflammatory phase of diabetic wounds contributes to abnormal myeloid cell function and delayed healing [257].

Adipocytes provide metabolic support for fibroblast proliferation and ECM remodeling during wound healing. The browning of WAT enhances fibroblast migration by increasing the production of neuregulin 4, a process diminished in diabetic wounds [258, 259]. Recent studies have explored the potential trans‐differentiation between adipocytes and myofibroblasts, but this remains a topic of debate due to conflicting findings [253, 254, 260].

### 4.9 Innervation

The role of cutaneous nerves in wound healing remains underexplored but is increasingly recognized as significant. Neurite outgrowth has been shown to enhance fibroblast differentiation into myofibroblasts, promoting stronger ECM contraction [261]. Schwann cells, the peripheral nerve glial cells, undergo de‐differentiation and proliferation after wounding, secreting various growth factors that influence inflammation and angiogenesis [262]. These nerve terminals and glial cells surround epidermal cells, regulating their differentiation and contributing to re‐epithelialization [263].

Neurotrophic factors also play a role in wound healing. For instance, neurotrophin‐3 is upregulated in the epidermis after injury and facilitates hyperinnervation [264]. Neural endings release neuropeptides like calcitonin gene‐related peptide, which promote immune cell recruitment and efferocytosis, enhancing skin and muscle injury repair [265, 266]. Other neuropeptides, such as substance P (SP), are also involved in wound healing, promoting angiogenesis and modulating macrophage phenotypes. However, the levels of these neuropeptides are reduced in DFUs, impairing their roles in angiogenesis and macrophage regulation [267].

These findings highlight the multifaceted contributions of innervation and neuropeptides to wound healing. In diabetic wounds, disrupted innervation and reduced neuropeptide levels contribute to delayed and impaired repair, underscoring the potential for therapeutic strategies targeting the nervous system to improve outcomes.

## 5 Animal Models for Research on Diabetic Wounds

In vitro models, such as 2D fibroblast cultures derived from DFU patients, provide valuable insights but lack the complexity of in vivo systems, which remain the gold standard for studying the biological processes underlying diabetic wounds. Animal wound models are classified based on the species used, methods of diabetes induction, and wound creation and assessment techniques. Ex vivo models also offer valuable perspectives and are considered in this discussion.

Diabetes induction in animal models varies by treatment, time window, diet, age, and sex, requiring careful interpretation when translating findings to humans. While DFUs occur in both type I and type II diabetes, their prevalence is higher in type II diabetes, likely due to differences in age, cumulative disease duration, and study design [70]. Body mass index is a significant risk factor for DFUs, with obesity strongly linked to insulin resistance and type II diabetes [268, 269]. Given that DFUs arise from long‐term hyperglycemia and systemic metabolic dysfunction, type II diabetes models are particularly relevant for studying diabetic wound healing.

### 5.1 Zebrafish Models

Nonmammalian models offer significant advantages for diabetic research, including short lifespans, rapid reproduction, and high‐throughput screening capabilities using whole‐genome RNA interference strategies [270]. Among nonmammalian models, zebrafish uniquely serve as an effective tool for studying wound healing in diabetic contexts. Their mammalian‐like ghrelin and leptin activity regulates lipid storage and metabolism, and their insulin‐producing cells share signaling pathways with mammals, making zebrafish suitable for mimicking diabetes [271, 272, 273]. Hyperglycemia, a hallmark of diabetes, can be induced in zebrafish by beta‐cell depletion [274].

Zebrafish skin shares structural similarities with human skin, including epidermis, blood vessels, and nerves. Their caudal fin provides a simple yet symmetrical model for wound repair, encompassing processes such as inflammation, granulation, and neovascularization [275]. Unlike mammals, zebrafish lack specialized immune cell subsets, such as T cells, while neutrophil‐like and macrophage‐like cells are present. Re‐epithelialization occurs as the first step of zebrafish wound healing, preceding inflammation [276, 277]. Another advantage is the transparency of juvenile zebrafish, enabling real‐time observation of wound repair through genetically engineered fluorescence varieties [276].

Hyperglycemia in zebrafish can be induced through chemical agents, dietary interventions, or a combination of both. Streptozotocin injections, such as weekly intraperitoneal doses of 0.35 mg/g or subcutaneous injections after a 4% cholesterol diet, induce prolonged hyperglycemia exceeding 300 mg/dL [278, 279]. Simpler methods involve immersing zebrafish in aqueous solutions containing inducing agents like alloxan or glucose, which can induce diabetes in just days [280, 281]. Skin wound models are easily constructed using skin biopsy needles or lasers [275, 276].

### 5.2 Rodent Models

Rodent models are widely used in biomedical research due to their physiological similarities to humans, rapid breeding, manageable size, and the availability of standardized methods and genetic tools [270]. These attributes make rodents particularly suitable for diabetic wound healing studies.

Diabetes in rodents can be induced through genetic engineering, diet, or chemical destruction of pancreatic β‐cells. Among genetic models, *ob/ob* mice (leptin‐deficient) and *db/db* mice (leptin receptor‐deficient) are commonly used. *ob/ob* mice exhibit early‐onset obesity with hyperphagia, low energy expenditure, and insulin resistance but only mild hyperglycemia. In contrast, *db/db* mice develop significant hyperglycemia (over 400 mg/dL), making them more representative of diabetes. Zucker rats (*fa/fa*), with defective leptin receptor translocation, are prone to obesity but less so to overt diabetes. Their diabetic variant, the Zucker diabetic fatty rat, is hyperglycemic due to β‐cell transcription defects inherited independently of leptin receptor mutations [270].

Diet‐induced obesity (DIO) models closely mimic the pathogenesis of human obesity and type II diabetes. C57BL/6J mice are widely used for DIO studies, exhibiting obesity, glucose intolerance, and insulin resistance with a high‐fat, high‐carbohydrate diet. However, hyperglycemia often requires additional interventions, such as low‐dose streptozotocin injections, to induce β‐cell damage [282]. In rats, Sprague–Dawley rats are the most common DIO model, with certain populations naturally developing obesity and metabolic dysfunction under high‐energy diets. Selective breeding has also produced Zucker Diabetic Sprague–Dawley rats, which develop diabetes slowly, better mimicking human type II diabetes while retaining functional leptin signaling [283].

The full‐thickness excisional wound model is the most widely used for studying diabetic wound healing [284]. This model involves removing skin layers, from the epidermis to the subcutaneous fascia, using biopsy punches or scissors. To study re‐epithelialization and granulation tissue formation, a silicon splint is often applied to minimize wound contraction, which is otherwise enabled by the panniculus carnosus or fascia, a thin muscle layer and a loose connective tissue layer beneath the subcutaneous fat in rodents. However, splinting is unnecessary when studying the role of subcutaneous fascia in wound healing. Alternatively, incisional models are employed to investigate surgical incision repair, while infected wound models incorporate microbiota to mimic infection. Other models, such as burn or ischemic wounds, have specific applications but rarely replicate the conditions of human DFUs [285].

Despite similarities in skin structure, notable differences exist between human and rodent skin. Human skin is thicker, firmer, and more adherent to underlying tissues, while rodent skin is thinner, looser, and easily separated with minimal force. Rodent wound healing relies heavily on rapid wound contraction, a process facilitated by the panniculus carnosus or the fascia, a subject of ongoing investigation. In contrast, human skin primarily heals through re‐epithelialization. Additional anatomical differences include the presence of epidermal rete ridges and deeper dermal papillae in humans, as well as the scarcity and uneven distribution of hair follicles. These disparities must be considered when translating findings from rodent models to human wound healing [286].

### 5.3 Large Animal Models

Pigs are the primary large animal model used in studies of diabetes and skin repair due to their closer biological and anatomical resemblance to humans compared with rodents or nonmammalian organisms. Porcine skin shares key similarities with human skin, including comparable thickness, well‐developed rete ridges, dermal papillae, and subdermal adipose tissue. Histological features, such as keratins, fibronectin, and vimentin staining, as well as the biochemical properties of dermal collagen, also align closely with human skin [287]. Importantly, partial thickness wound closure in pigs follows a re‐epithelialization pattern similar to that observed in humans [288].

Pigs offer advantages such as a larger skin surface, allowing multiple wound sites on the back, and the ability to perform delicate surgical procedures. However, they require significant resources, including skilled veterinary care for anesthesia, large breeding spaces, and higher maintenance costs. While DIO alone cannot induce diabetes in pigs, diabetes can be established using streptozotocin injections or through transgenic approaches. Transgenic diabetic pigs have been developed to express dominant‐negative glucose‐dependent insulinotropic polypeptide receptors, mutant insulin, or hepatocyte nuclear factor 1α mutations [289, 290, 291]. Additionally, excessive dietary energy intake, supplemented with fat or fructose, can induce overweight conditions.

Beyond pigs, primates have also been used in diabetic wound research. In one study, baboons were employed to investigate incisional wound healing under diabetic conditions [292]. Diabetes was induced using intravenous STZ, followed by daily insulin injections for 3 months to achieve metabolic control comparable to human patients [293].

Despite fundamental differences in skin anatomy and wound healing physiology between humans and rodents, a key limitation lies in the inability of rodent models to fully recapitulate the complex pathophysiology of human DFUs. Human DFUs typically arise after prolonged duration of diabetes (usually decades) and are compounded by DPN. In contrast, DIO models of type II diabetes exhibit temporal and variable disease duration (8–18 weeks), which may not adequately recapitulate the chronic neuropathic progression seen clinically [294, 295]. Moreover, standard dorsal excisional wound models in mice fail to stimulate pressure‐induced ulcer progressions. Unlike human plantar surfaces, which experience sustained mechanical loading during walking, murine dorsal skin is nonweight bearing. These differences prevent accurate modeling of the dynamic interplay between pressure, ischemia, and microtrauma, which drives ulcer development in neuropathic patients. Consequently, these models limit the translational relevance of interventions targeting pressure modulation and off‐loading strategies.

## 6 Emerging Therapeutic Methods

Preventing the occurrence of DFUs in diabetic patients involves daily foot inspection, proper hygiene, moisturization, well‐fitting footwear, and maintaining a healthy lifestyle. Regular follow‐ups with podiatrists are essential to assess foot pressure and perfusion, while optimal blood glucose and lipid control help mitigate complications in the vascular and nervous systems. For patients with DFUs, surgical management is critical to ensure healing. Debridement, a standardized approach, removes devitalized tissue and infected areas to facilitate healing. Proper wound dressings, designed to maintain a suitable microenvironment for tissue growth, re‐epithelialization, and drainage, along with appropriate antibiotics, prevent infection recurrence. Advanced therapies like negative pressure wound therapy and hyperbaric oxygen therapy are also recommended. Additionally, reducing repetitive mechanical stress on the sole and involving a multidisciplinary team, including podiatrists, endocrinologists, and dietitians, ensure timely recognition, early intervention, and cooperative management to prevent recurrence or complications.

Despite ongoing efforts, current traditional therapeutic combinations often fail to achieve rapid and effective healing of DFUs. These strategies, typically administered through routine protocols, face significant limitations in achieving consistent clinical outcomes, primarily due to profound cellular dysfunction resulting from long‐term metabolic imbalance. Furthermore, compromised tissue perfusion severely restricts targeted drug delivery, reducing systemic bioavailability. Consequently, next‐generation therapies must focus on restoring cellular functions while improving the local bioavailability of therapeutic agents in the exudative wound microenvironment.

Emerging therapies, such as the application of growth factors, bioengineered skin substitutes, targeted inhibitors, and stem cell‐based therapies, represent significant advancements in the field. Recent clinical trials evaluating these novel approaches are summarized in Table 1. By addressing both wound healing and the underlying cellular dysfunctions, these innovative therapies offer renewed hope for reducing complications and improving the quality of life for individuals with diabetes.

**TABLE 1:** Clinical trials of emerging treatments for diabetic foot ulcers.

Therapy	Study ID	Interventions	Country	Registration date	Phase
Growth factors	CTRI/2024/09/073475	Composition of nanopolymers and growth factors versus EUSOL‐soaked gauze as standard wound dressing method	India	05/09/2024	N/A
	CTRI/2024/06/068922	Application of recombinant human epidermal growth factor versus normal saline dressing	India	14/06/2024	4
	CTRI/2024/05/067239	Application of recombinant human epidermal growth factor versus conventional saline dressing	India	13/05/2024	3
	CTIS2023‐504275‐24‐01	Application of plasma rich in growth factors	Spain	27/02/2024	4
	CTRI/2023/03/050481	Application of autologous growth factor versus dressing as per the standard of care	India	09/03/2023	N/A
	IRCT20120215009014N352	Application of dry amniotic dressing, dressing with platelet‐derived growth factor or conventional surgical debridement	Iran	21/04/2020	2
	EUCTR2018‐001724‐19‐BG	Application of recombinant human mutant nerve growth factor painless versus placebo	Bulgaria	08/08/2018	1/2
	NCT01629199	Application of recombinant human epidermal growth factor versus placebo	Korea	25/06/2012	3
	RPCEC00000048	Application of recombinant human epidermal growth factor versus placebo	Cuba	24/12/2010	2
	EUCTR2010‐021015‐16‐GB, EUCTR2010‐021014‐33‐HU	Application of trafermin (recombinant human basic fibroblast growth factor) 0.01% spray versus placebo	Belgium, Denmark, Germany, Hungary, Netherlands, Sweden, United Kingdom, Czech Republic, France, Hungary, Italy	09/08/2010, 06/08/2010	3
Stem cell and exosomes	IRCT20240513061768N1	Application of silicone dressing along with alginate containing placental‐derived stem cells or whose secretome versus silicone dressing only	Iran	25/05/2024	2
	NCT06373809	Transplantation of placenta‐derived stem cells	Nicaragua	08/04/2024	1
	NCT06319287	Application of topical purified exosome product‐TISSEEL	United States	13/03/2024	2
	NCT06231771	Application of allogeneic mesenchymal stem cells	Not provided	21/01/2024	1/2
	KCT0009122	Subcutaneously injection of bone marrow‐derived stem cell concentrate and application of allogeneic dermal matrix with polyurethane foam dressing versus application of allogeneic dermal matrix with polyurethane foam dressing only	Korea	19/01/2024	N/A
	IRCT20131009014957N16	Application of secretome spray or hydrogel–secretome composite derived from human mesenchymal stem cells	Iran	12/10/2023	1/2
	IRCT20191007045008N2	Application of human placenta‐derived mesenchymal stem cells with acellular amniotic membrane (AMM) graft versus AMM only	Iran	26/07/2023	2
	NCT05595681	Application of adipose tissue‐derived mesenchymal stromal/stem cells	Denmark	23/09/2022	1
	IRCT20180619040147N5	Local injection of menstrual blood‐derived mesenchymal stem cells	Iran	29/03/2021	3
	IRCT20191007045008N1	Application of placenta scaffold containing adipose‐derived mesenchymal stem cells versus scaffold only, or stem cell only or standard dressing	Iran	17/01/2020	1/2
	IRCT20190214042712N1	Application of adipose‐derived mesenchymal stem cells loaded on nanofiber scaffolds versus routine treatments	Iran	17/09/2019	N/A
	IRCT20170612034485N2	Application of allogenic mesenchymal stem cells	Iran	11/02/2018	1/2
	NCT03259217	Transplantation of mesenchymal stem cells seeded in chitosan scaffold	Not provided	21/08/2017	1
	EUCTR2016‐004109‐15‐PL	Application of autologous adipose‐derived mesenchymal stem cells	Poland	10/05/2017	2
	EUCTR2017‐000234‐57‐DE	Application of allogeneic skin‐derived ABCB5‐positive mesenchymal stem cells	Germany	13/03/2017	1
	EUCTR2015‐005580‐16‐DK	Application of ex vitro cultured human mesenchymal stem cells	Denmark	09/06/2016	1
	ACTRN12616000442404	Application of stem/progenitor cells	Australia	06/04/2016	N/A
	CTRI/2015/01/005355	Intramuscular injection of autologous bone marrow‐derived progenitor cell therapy versus intravenous injection of prostaglandins	India	05/01/2015	1/2
	EUCTR2012‐001825‐28‐CZ	Application of autologous bone marrow aspirate concentrate	Czech Republic	20/08/2012	3
	NCT01065337	Application of expanded autologous bone marrow stem cells	Germany	08/02/2010	2
MMPs	TCTR20230502001	Consumption of golden rice cookies versus conventional low sugar cookies	Indonesia	02/05/2023	0
Skin substitutes	CTRI/2024/11/076815	Application of 3D printed skin versus autologous skin graft	India	14/11/2024	N/A
	NCT06557122	Application of advanced bioengineered skin substitute	United States	12/08/2024	N/A
	NCT06470087	Application of type‐I collagen‐based skin substitute versus human amnion/chorion membrane	India	31/05/2024	N/A
	NCT06449638	Application of multiple human placental‐based skin substitutes	United States	28/05/2024	N/A
	NCT06384183	Application of Kerecis fish skin graft	United States	22/04/2024	N/A
	IRCT20080728001031N33	Application of bi‐layered allogeneic cultured keratinocyte and fibroblast skin substitute	Iran	03/07/2022	1
	NCT04927702	Application of living cellular skin substitute or synthetic hybrid‐scale fiber matrix versus standard care	United States	03/06/2021	N/A
	KCT0004373	Application of micronized acellular dermal matrix with negative pressure wound therapy (NPWT) versus NPWT only	Korea	23/10/2019	4
	ChiCTR1900026711	Application of acellular allogeneic dermis composite autologous skin graft versus conventional autologous skin graft	China	19/10/2019	0
	NCT04133493	Application of omega3 wound fish skin graft	United States	08/10/2019	N/A
	ISRCTN64926597	Application of decellularized dermal allograft	United Kingdom	06/06/2017	N/A
	NCT02870816	Application of decellularized dermal allograft	United States	14/08/2016	N/A
	NCT02070835	Application of tissue engineered skin substitute versus amniotic membrane wound graft	China	22/02/2014	N/A
	NCT01676272	Application of bioengineered skin substitute versus human skin allograft	United States	28/08/2012	N/A
	NL‐OMON30845	Application of autologous tissue engineered skin substitute	Netherlands	13/11/2007	N/A

*Data source*: International Clinical Trials Registry Platform (https://trialsearch.who.int/), accessed on May 14, 2025.

### 6.1 Supplement of Growth Factors

The topical application of growth factors represents a transformative approach in managing DFUs, aiming to enhance wound healing by promoting cellular proliferation, angiogenesis, and collagen synthesis. Delivered via advanced systems such as hydrogels, sprays, and impregnated dressings, growth factors significantly improve healing rates and outcomes by directly addressing the cellular dysfunctions in diabetic wounds. FGFs consist of 23 family members in humans and play key roles in wound healing and metabolic regulation [296]. Endocrine FGFs influence carbohydrate and lipid metabolism, while paracrine FGFs drive processes like angiogenesis, fibroblast proliferation, and granulation tissue formation [297, 298]. FGFs delivered through carriers like hydrogels, lipid nanoparticles (LNPs), or coacervates accelerate wound healing by enhancing re‐epithelialization, ECM deposition, and angiogenesis [299, 300, 301, 302]. FGF1 mitigates oxidative stress via the SIRT1–Nrf2 pathway and increases HO‐1 levels [303]. FGF2 promotes diabetic wound healing through HIF‐1 activation in endothelial cells [304], while FGF21 restores endothelial function by inhibiting NF‐κB signaling and NLRP3 inflammasome activation [305].

VEGFs, essential for angiogenesis, are upregulated during wound healing through hypoxia‐induced HIF‐1 signaling [306]. VEGFs promote vasodilation, endothelial migration, and proliferation, facilitating granulation tissue formation. Recombinant human VEGF has been approved by United States Food and Drug Administration [307]. It has been shown to accelerate diabetic wound healing and to resolve inflammation also by repolarizing macrophages [308]. To address VEGF instability, innovative delivery systems like LNPs encapsulating circular RNA encoding VEGF or materials with VEGF‐overexpressing HUVECs have been developed. These approaches enhance angiogenesis, collagen deposition, and mitochondrial protection in high‐glucose conditions [309, 310].

PDGFs stimulate inflammatory cell chemotaxis and fibroblast migration to the wound site. Clinical trials have shown that recombinant human PDGF accelerates diabetic wound closure and reduces amputation rates [311, 312]. Controlled‐release systems improve PDGF stability in the proteolytic environment of DFUs [313]. However, safety concerns regarding malignancy risks prompted the development of engineered PDGF variants with extended tissue retention and reduced systemic leakage, eliminating tumor‐promoting effects while enhancing wound repair [314, 315].

EGFs, derived from fibroblasts, platelets, and macrophages, promote keratinocyte proliferation and migration, facilitating re‐epithelialization. Reduced EGF levels and receptor dysfunction in DFUs impair related healing pathways [306]. Recombinant human EGF has demonstrated faster and more complete ulcer healing in clinical trials compared with placebo [316, 317].

The application of growth factors holds great promise for DFU treatment by replenishing deficiencies and exhibiting prohealing effects, such as promoting angiogenesis, proliferation, and ECM deposition. However, individual responses to therapies vary, underscoring the need for personalized approaches to dosage and administration. Current growth factor products are expensive, imposing financial burdens due to daily usage. To mitigate these costs, advanced delivery vehicles that prevent degradation and ensure controlled release are needed. Innovations like 3D bioprinting may enable the creation of growth factor‐enriched scaffolds for enhanced tissue regeneration. Continued research will likely unlock new and cost‐effective strategies to improve diabetic wound healing.

### 6.2 Application of Stem Cells and Exosomes

Mesenchymal stem cells hold significant promise in diabetic wound healing due to their regenerative and immunomodulatory properties [143, 318, 319]. Exosomes, lipid‐bilayer vesicles containing proteins, nucleic acids, and lipids, facilitate intercellular communication and deliver therapeutic cargo without significant immunogenicity or cytotoxicity. Their stability, ease of engineering, and ability to penetrate biological barriers make them ideal for drug delivery and wound healing applications [320, 321].

Adipose‐derived stem cells (ADSCs) and their exosomes (ADSC‐Exo) promote immune regulation, ECM remodeling, angiogenesis, and fibroblast proliferation [322, 323]. ADSC‐Exo, often delivered via hydrogels or LNPs, enhance diabetic wound healing by regulating HIF‐1α signaling, oxidative stress, and the AKT axis. Strategies like hypoxia pretreatment or genetic modification (e.g., overexpressing HIF‐1α or delivering specific noncoding RNAs) further amplify their therapeutic potential [324, 325, 326, 327, 328, 329, 330].

Bone marrow‐derived stem cell exosomes (BMSC‐Exo) also improve diabetic wound healing through various mechanisms, including ferroptosis inhibition, macrophage polarization to the M2 phenotype, and keratinocyte activation via pathways such as PI3K/AKT and Nrf2. Pretreatment under conditions like hypoxia or with agents such as pioglitazone or IFN‐γ enhances their efficacy, while genetic modifications (e.g., HO‐1 or MG53 overexpression) improve endothelial cell repair and angiogenesis [331, 332, 333, 334, 335, 336, 337, 338, 339, 340, 341]. Engineered BMSC‐Exo enriched with miRNAs (e.g., miR‐140‐3p, miR‐132‐3p) enhance regeneration of deep tissue layers and facilitate multifactorial repair in diabetic wounds [342, 343].

Human umbilical cord mesenchymal stem cells and their exosomes have been investigated in clinical trials, demonstrating clinical benefits for DFU healing by promoting endothelial repair, macrophage polarization, and ECM deposition [344]. Pretreatment with Wnt pathway agonists or Coenzyme Q10 enhances their efficacy by upregulating miRNAs and prohealing proteins [345, 346, 347, 348, 349, 350]. MSCs from other sources, such as placental, amniotic, or dermal (ABCB5^+^), and their exosomes also contribute to angiogenesis and immune regulation, promoting repair of chronic nonhealing wounds [351, 352, 353].

### 6.3 Targeting MMPs

MMPs, particularly MMP9, play complex roles in diabetic wound healing, often leading to impaired repair. Targeting MMPs or modulating their activity has shown therapeutic potential in managing DFUs [354]. For instance, direct inhibition of MMP9 using selective inhibitors, such as ND‐322 and ND‐336, has been demonstrated to accelerate wound healing in diabetic models [227, 355, 356]. Similarly, l‐carnosine combined with curcumin inactivates MMP9 by chelating its zinc active center, reducing oxidative stress and inflammation [357]. RNA‐based approaches, such as siRNA targeting MMP9, delivered via advanced systems, offer sustained inhibition without toxicity, promoting healing by regulating MMP9 levels [358, 359, 360, 361].

Innovative drug delivery systems responsive to MMP‐rich microenvironment in diabetic wounds have also been developed. These include MMP‐responsive hydrogels and microspheres, which release therapeutic agents like ADSC‐derived exosomes, curcumin analogues, or deferoxamine in response to MMP activity [326, 362, 363, 364]. Smart dressing systems incorporating precise MMP9 sensors further enhance localized treatment by enabling controlled release of antimicrobial agents like silver nanoparticles while monitoring wound conditions [365].

In addition to specific inhibitors, natural compounds, micronutrients, and gases have been shown to regulate MMP activity broadly. Quercetin glucuronide promotes MMP1 expression while reducing MMP9 levels, and berberine increases TIMP1 expression while downregulating MMP9 via TrxR1 activation and JNK inhibition [366, 367]. Other interventions, including vitamin D, autologous platelet‐rich gels, NO donors, and hyperbaric oxygen, reduce activities of MMPs while promoting wound healing [368, 369, 370, 371]. Intriguingly, certain microbiota, such as *Alcaligenes faecalis*, can restore MMP balance, enhancing re‐epithelialization and wound repair [372]. These findings highlight the potential of MMP‐targeting strategies for DFUs, offering diverse therapeutic options tailored to individual MMP profiles.

The secretion of MMPs is one crucial characteristic of senescence‐associated secretory phenotype, which suggests that cellular senescence is also one of the pivotal contributors to the chronicity of DFUs, driven by hyperglycemia, inflammation, and oxidative stress, and that targeting senescent cells or their secretory phenotype offers a promising strategy to break the cycle of impaired healing [373]. Recent studies provided several possible solutions as the senomorphic strategies to refresh the cellular component and promote homeostasis and repair. IL‐11 has been newly recognized as key regulator in inflammatory and aging related disease, which might serve as a promising target in future therapeutic design [374, 375].

### 6.4 Targeting Epigenetic Modifications

Epigenetic changes, including DNA methylation, histone modification, and RNA methylation, play significant roles in the pathogenesis of diabetes and DFUs by disrupting proper gene regulation during wound healing [376, 377]. These modifications hinder timely expression of critical genes, impair cellular functions, and delay repair. Emerging therapeutic approaches targeting these modifications show promise for improving clinical outcomes in DFUs.

DNA methylation regulates gene expression by influencing promoter accessibility. In diabetic wounds, hyperglycemia‐induced upregulation of DNMT1 leads to hypermethylation of promoters for critical genes like Ang‐1, impairing endothelial function. Inhibition of DNMT1 with compounds such as 5‐aza‐deoxycytidine restores Ang‐1 expression and promotes healing [378]. Similarly, pterostilbene reverses hypermethylation‐related silencing of Nrf2, a key regulator of oxidative stress, in endothelial cells [379]. Conversely, hypomethylation of promoters due to reduced DNMT activity disrupts perfusion, and supplementation with methyl donors has been shown to improve blood flow and repair in diabetic wounds [380]. In addition, Tet methylcytosine dioxygenase 2 and thymine DNA glycosylase‐mediated DNA demethylation exacerbates inflammation in diabetic wounds by promoting MMP9 overexpression, while inhibition of this pathway offers a potential therapeutic avenue for reducing inflammation and promoting proper wound healing [381, 382, 383].

Histone modifications, such as methylation and acetylation, regulate chromatin accessibility and transcription. Histone methylation affects the interaction between nucleosome and transcriptional factors, while acetylation unwinds packed DNA for the accessibility to expression. In diabetic wounds, dysregulated histone methyltransferases like MLL1 and JMJD3 skew macrophage activity toward prolonged inflammation. MLL1 enhances TLR4 expression in macrophages by catalyzing H3K4 methylation at the TLR4 promoter, contributing to sustained inflammatory responses. Diabetic monocytes exhibit elevated MLL1 levels, and prediabetic macrophages treated with MLL1 inhibitors show reduced inflammation, suggesting a therapeutic potential in targeting MLL1 for diabetic wound healing [384, 385]. Similarly, JMJD3 demethylates H3K27, enhancing NF‐κB signaling and promoting chronic inflammatory responses. Inhibition of JMJD3 in macrophages has been shown to reduce late‐stage inflammation and improve wound healing [294, 386]. In contrast, the histone methyltransferase Setdb2 is downregulated in diabetic macrophages. This enzyme mediates H3K9 trimethylation at NF‐κB binding sites on inflammatory cytokine promoters, thereby suppressing their transcription and facilitating macrophage phenotypic transition [387].

Histone acetyltransferase MOF, upregulated in diabetic wounds, drives inflammation through H4K16 acetylation and NF‐κB activation, while MOF deletion or TNF‐α inhibitors mitigate excessive inflammation and accelerate wound healing in DIO mice [388]. Similarly, histone deacetylases like HDAC3 and HDAC4 are overexpressed in diabetic wounds, perpetuating inflammation. Pharmacological inhibitors targeting HDACs reduce inflammation and enhance re‐epithelialization, positioning HDAC inhibition as a promising therapeutic strategy for DFUs [389, 390, 391, 392, 393].

RNA modifications, such as N6‐methyladenosine (m6A) modification, regulate mRNA stability and translation, with dysregulation in diabetic wounds linked to mitochondrial dysfunction and impaired repair. METTL3, a key m6A writer, is downregulated in diabetic endothelial cells, reducing the expression of survival factors like NDUFB5. Overexpression of METTL3 restores mitochondrial homeostasis and promotes angiogenesis, suggesting its therapeutic potential [394, 395, 396]. Similarly, YTHDC1, an m6A reader, supports autophagic flux in keratinocytes, while its downregulation in hyperglycemia exacerbates cellular dysfunction. Enhancing YTHDC1 expression could provide a novel strategy for improving keratinocyte behavior and wound repair [397, 398].

Targeting epigenetic modifications offers a compelling strategy for DFUs, addressing the underlying causes of delayed healing by restoring proper gene expression and cellular function. Approaches like DNMT inhibition, histone modification regulation, and m6A modulation have demonstrated efficacy in preclinical models, paving the way for translational applications. Future research should focus on optimizing delivery systems and identifying safe and effective epigenetic modulators for clinical use.

### 6.5 Bioengineered Skin Substitutes

Skin substitutes encompass a diverse range of biological, synthetic, or hybrid biosynthetic materials engineered to facilitate wound healing by mimicking the structure and function of native skin and delivering bioactive components. Acting as transformative therapeutic tools, they serve as temporary or permanent skin replacements. These advanced biomaterials promote re‐epithelialization, enhance angiogenesis, and modulate inflammatory responses, thereby restoring the wound microenvironment and supporting wound repair [399, 400]. In the management of DFUs, skin substitutes demonstrate immense therapeutic value and application potential. Ideally, effective substitutes should fulfill key criteria, including nonimmunogenicity, biocompatibility, regenerative capacity, protective functionality, nonpathogenicity, and long‐term durability [22].

Skin substitutes can be classified according to their intended duration of use, composition, and functional attributes. Temporary substitutes, typically composed of one or two layers, primarily act as dressings to protect the wound surface against secondary mechanical injury, bacterial colonization, and fluid evaporation [401]. In contrast, more durable or permanent substitutes possess enhanced capabilities aimed at supporting epithelial or dermal reconstruction. Epidermal substitutes, usually comprising cultured keratinocyte autografts formed into thin layers, contribute to re‐epithelialization; however, they are often vulnerable to frictional forces due to the absence of dermal–epidermal junction [402]. Dermal substitutes, commonly fabricated from cell‐free biomaterials, primarily serve as three‐dimensional scaffolds that guide fibroblasts and endothelial cell migration and infiltration, creating a dynamic microenvironment favorable for tissue regeneration. Some dermal substitutes incorporate cellular components, such as fibroblasts or MSCs, to accelerate stromal matrix deposition and wound repair. Advanced dermo‐epidermal constructs are generated through sophisticated bioengineering strategies, producing keratinocytes‐attached, fibroblast‐laden, and cytokine‐enriched synthetic skin, which requires precise 3D construction to recapitulate native cytoarchitecture of skin [22, 403, 404].

3D skin equivalents highlight the importance of proper skin layer stratification. Techniques such as bioprinting have significantly enhanced the proliferation and migration of various cell types, leading to improved healing outcomes [402, 405]. Early methods established skin‐like structures through layer‐by‐layer cocultures of keratinocytes and fibroblasts, often raised at an air–liquid interface to promote stratification [405]. To further support skin layer formation, natural polymers and collagen‐based hydrogels have been employed as scaffolding materials. More advanced bioprinting techniques enable precise spatial arrangement of living cells and ECM components. Guided by computer‐aided design, fibroblasts, keratinocytes, cytokines, and matrix can be printed in a multilayered fashion, even allowing for the incorporation of pigmentation features. Moreover, in situ skin bioprinting has emerged as a cutting‐edge approach, enabling individualized reconstruction of skin layers directly at the wound site [406]. By integrating with host tissues and adapting to wound‐specific needs, bioengineered skin substitutes represent a paradigm shift in diabetic wound care, bridging the gap between conventional therapies and functional tissue restoration.

However, profound inter‐patient heterogeneity, manifested through variations in wound etiology (neuropathic or ischemic), genetic predispositions affecting cellular responses and metabolic conditions might undermine the efficacy of mono‐therapeutic approaches targeting isolated pathways, as molecular targets fluctuate temporally and compensatory mechanisms emerge. These biological complexities are compounded by translational gaps: preclinical models inadequately recapitulate human comorbidities and chronicity timelines, while clinical trials suffer from inconsistent endpoint definitions and insufficient patient stratification. Collectively, these factors explain the divergent actual performance of mechanistically promising therapies, underscoring the necessity for precision medicine frameworks integrating patient‐specific pathophysiology.

Another critical consideration is the substantial cost of bioactive therapeutics. Production and storage complexities of biologics such as inhibitors or growth factors significantly raise treatment expenses. Without precision guidance tailored to individualized systemic profiles and wound conditions, indiscriminate application may lead to considerable resource waste. These economic constraints highlight the urgent need for personalized assessment protocols and evidence‐based guidelines to improve cost‐effectiveness in DFU management.

Based on the imperative for precision medicine frameworks, future research should aim to resolve key knowledge gaps by integrating multidimensional profile, such as single cell transcriptomics and metabolomics, with high‐resolution intravital imaging or real‐time sensing. This approach will allow researchers to capture dynamic cellular responses and uncover novel therapeutic targets within dysfunctional signaling networks. Importantly, translating these findings will require precision medicine pipelines that stratify patients based on deep molecular phenotyping. Such strategies promise to shift diabetic wound care from generalized protocols toward adaptive interventions tailored to each patient's unique cellular and molecular landscape.

## 7 Conclusions and Perspectives

DFUs remain one of the most severe complications of diabetes, garnering increasing attention due to their high prevalence, low healing rates, and significant risks of infection, recurrence, and amputation. As the diabetic population grows, the burden continues to increase for both individuals and healthcare systems. The development of DFUs often arises from combined pathological preconditions, such as peripheral neuropathy, vascular diseases, and skin defects, which increase susceptibility to repetitive minor injuries and microbiological contamination. Compounding these factors, the diabetic microenvironment, marked by hyperglycemia, dyslipidemia, and oxidative stress, dysregulates cellular functions critical for healing. This interplay leads to persistent yet insufficient inflammation, impaired stromal filling, reduced angiogenesis, and delayed re‐epithelialization, ultimately contributing to poor wound closure. While significant progress has been made in understanding these mechanisms, much remains to be explored, particularly at the molecular and cellular levels.

Animal models have been instrumental in advancing diabetic wound research. Rodents, widely used due to their physiological similarities to humans and ease of handling, dominate the field, while large animal models like pigs offer a closer anatomical resemblance. Primate models, although highly relevant, remain underutilized due to ethical constraints, high costs, and extended study duration. To gain deeper insights, future research should leverage integrative technologies like single‐cell RNA sequencing to compare human samples with findings from animal models. Such approaches could unravel conserved pathological processes and improve the translational potential of preclinical studies. Traditional 2D culture systems, often used to study cellular behavior in the diabetic microenvironment, have limitations, such as loss of in situ cell characteristics due to stiff culture surfaces. Advanced 3D organoid models and explant cultures could serve as promising alternatives, more faithfully replicating the complexity than 2D systems.

Despite the availability of clinical interventions, the treatment of DFUs remains suboptimal. Emerging therapies, such as growth factors, stem cells, exosomes, MMP modulators, epigenetic interventions, and skin substitute application, offer hope for more effective outcomes. However, inconsistencies in therapeutic efficacy often arise from the dynamic nature of wound healing phases, where the timing of intervention significantly influences results. For instance, earlier views emphasized excessive inflammation in nonhealing wounds, while recent evidence highlights insufficient early inflammation coupled with prolonged persistence. Despite promising efficacy demonstrated by numerous novel therapeutics and dressings in diabetic animal models, their clinical translation rates remain disappointingly low. This translational bottleneck stems from fundamental disparities between preclinical models and human patients: animal studies exhibit phenotypic homogeneity, whereas human DFUs manifest as highly heterogeneous pathological entities encompassing dynamic variables such as wound chronicity duration, severity of comorbid endocrinopathies, and metabolic dysregulation magnitudes. Such multifaceted complexity fundamentally resists modulation through monotherapeutic strategies. These acknowledgements underscore the need for personalized assessment and treatment strategies tailored to the unique conditions of each patient's ulcer, including nerve and vascular health, inflammation, and infection status.

Standardized clinical wound evaluations, such as the WIFI (wound, ischemia, foot infection) classification system, provide valuable tools for assessing DFU severity. However, these assessments lack cellular‐ or molecular‐level resolution, which fundamentally impedes the implementation of personalized treatment regimens tailored to dynamic patient‐specific wound states and spatially heterogeneous cellular phenotypes. A comprehensive molecular classification system could enhance DFU management by incorporating key metrics such as skin structure integrity (epidermis, dermis, fat pad, and fascia), inflammatory markers (cytokines and cell infiltration ratios), metabolic parameters (glucose, SFAs, and ROS concentrations), infection status, enzymatic profiles and even single‐cell level omics. These data, derived from multiomics approaches and clinical tests, could guide the development of tailored therapeutic combinations and resolve the fundamental questions, such as the cell‐specific metabolic reprogramming dynamics during diabetic wound healing phases, the role of dysregulated intercellular communication and the spatial heterogeneity of metabolic microenvironment. Thus, there is a pressing need to implement precision diagnostic frameworks that combine molecular profiling with clinical parameters. Clinical trials play a pivotal role in validating these emerging classifications and correlating them with treatment efficacy. To advance this goal, it is essential to comprehensively integrate clinical data and systematically document therapeutic responses across diverse DFU patient cohorts. Such efforts will enable predictive modeling of treatment sensitivity based on multidimensional clinical metrics, ultimately guiding individualized management strategies, reducing amputation rates, and improving patient outcomes. As research continues to evolve, integrating innovative therapies with molecular‐level diagnostics offers an exciting path forward, potentially transforming the landscape of DFU treatment and significantly improving quality of life for patients with diabetes.

## Author Contributions

Writing—original draft preparation: R.W. and D.J. Writing—review and editing: R.W., S.G., Y.H.K., A.L., H.L., and D.J. Funding acquisition: H.L. and D.J. All authors have read and approved the final manuscript.

## Conflicts of Interest

The authors declare no conflicts of interests.

## Ethics Statement

The authors have nothing to report.

## Data Availability

The authors have nothing to report.
